# Supplementary material for: Within-sibship genome-wide association analyses decrease bias in estimates of direct genetic effects
Source: Nat Genet. 2022 May 9;54(5):581–92. doi: 10.1038/s41588-022-01062-7 (PMC9110300; doi:10.1038/s41588-022-01062-7)
Supplement: Supplementary file 1 — Supplementary information—consortia, funding, cohort descriptions, phenotype definitions and supplementary methods. [file 41588_2022_1062_MOESM1_ESM.pdf]

nature genetics

SUPPLEMENTARY INFORMATION

https://doi.org/10.1038/s41588-022-01062-7

In the format provided by the authors and unedited.

OPEN

Within-sibship genome-wide association analyses decrease bias in estimates of direct genetic effects

In the format provided by the authors and unedited

Supplementary Materials: Within-sibship genome-wide association analyses decrease bias in estimates of direct genetic effects

LJ Howe et al 2021

Table of Contents

Consortia 2

Funding information 3

Cohort descriptions 5

Phenotype definitions 32

Supplementary Methods 38

Consortia

Members of the Social Science Genetic Association Consortium (SSGAC)

Daniel J. Benjamin, David Cesarini, *Philipp Koellinger*, *Patrick Turley*, Aysu Okbay, Alexander I. Young, Michelle Meyer, Jonathan Beauchamp, Richard Karlsson Linnér, Fleur Meddens, Cornelius A. Rietveld, Ronald de Vlaming, Raymond Walters, Casper Burik, *Hermon Kweon*, Samantha Cherney, Chelsea Watson, Michael Bennett, Tami Gjorgjieva, Grant Goldman, Hariharan Jayashankar, Seyed Moeen Nehzati, Nancy Wang.

Italics denotes co-authorship on the manuscript.

Members of the Within-Family Consortium (WFC)

Laurence J Howe, Michel G Nivard, Tim T Morris, Ailin F Hansen, Humaira Rasheed, Yoonsu Cho, Geetha Chittoor, Rafael Ahlskog, Penelope A Lind, Teemu Palviainen, Matthijs D van der Zee, Rosa Cheesman, Massimo Mangino, Yunzhang Wang, Shuai Li, Lucija Klaric, Scott M Ratliff, Lawrence F Bielak, Marianne Nygaard, Alexandros Giannelis, Emily A Willoughby, Chandra A Reynolds, Jared V Balbona, Ole A. Andreassen, Helga Ask, Dorret I Boomsma, Archie Campbell, Harry Campbell, Zhengming Chen, Paraskevi Christofidou, Elizabeth Corfield, Christina C Dahm, Deepika R Dokuru, Luke M Evans, Eco JC de Geus, Sudheer Giddaluru, Scott D Gordon, K. Paige Harden, W. David Hill, Amanda Hughes, Shona M Kerr, Yongkang Kim, Antti Latvala, Deborah A Lawlor, Liming Li, Kuang Lin, Per Magnus, Patrik KE Magnusson, Travis T Mallard, Pekka Martikainen, Melinda C Mills, Pål Rasmus Njølstad, Nancy L Pedersen, David J Porteous, Karri Silventoinen, Melissa C Southey, Camilla Stoltenberg, Elliot M Tucker-Drob, Margaret J Wright, John K Hewitt, Matthew C Keller, Michael C Stallings, James J Lee, Kaare Christensen, Sharon LR Kardia, Patricia A Peyser, Jennifer A Smith, James F Wilson, John L Hopper, Sara Hägg, Tim D Spector, Jean-Baptiste Pingault, Robert Plomin, Alexandra Havdahl, Meike Bartels, Nicholas G Martin, Sven Oskarsson, Anne E Justice, Iona Y Millwood, Kristian Hveem, Øyvind Naess, Cristen J Willer, Bjørn Olav Åsvold, Jaakko Kaprio, Sarah E Medland, Robin G Walters, David M Evans, George Davey Smith, Caroline Hayward, Ben Brumpton, Gibran Hemani, Neil M Davies, Elham Assary, Michel Boivin, Tom A Bond, Mara Brendgen, Laurie Hannigan, Laura D Howe, William G Iacono, Matt McGue, Marcus R Munafò, Michael C Neale, Juan R Ordoñana, Hannah M Sallis, Eleanor Sanderson, Fartein A Torvik, Nicole M Warrington, Eivind Ystrøm.

Italics denotes co-authorship on the manuscript.

Funding information

LJH, TTM, YC, DAL, GDS, GH and NMD work in a unit that receives support from the University of Bristol and the UK Medical Research Council (MC_UU_00011/1 & 6). NMD is supported by a Norwegian Research Council Grant number 295989. GH is supported by the Wellcome Trust and Royal Society [208806/Z/17/Z]. This work was supported by the Cohorts for Heart and Aging Research in Genomic Epidemiology (CHARGE) National Heart, Lung, and Blood Institute (NHLBI) grant R01HL105756-09. MGN is supported by ZonMW grants 849200011 and 531003014 from The Netherlands Organisation for Health Research and Development, a VENI grant awarded by NWO (VI.Veni.191G.030) on NIH grant R01MH120219 and a Jacobs foundation research fellowship. DAL is PI of the Bristol British Heart Foundation Accelerator Award (AA/18/7/34219), is a British Heart Foundation Chair and National Institute of Health Research Senior Investigator (NF-0616-10102). JK receives support from Academy of Finland grants (#308248 & 312073). EMTD and KPH receive support from NIH grants (R01HD083613, R01HD092548). LK receives support from a RCUK Innovation Fellowship from the National Productivity Investment Fund (MR/R026408/1). SMK and JFW work in a unit that receives support from the UK Medical Research Council (MC_UU_00007/10). DIB receives support from a Royal Netherlands Academy of Science Professor Award (PAH/6635). DJB receives support from NIA/NIH grants R24-AG065184 and R01-AG042568 to UCLA and R56-AG058726 to the University of Southern California, Open Philanthropy (010623-00001), Ragnar Söderberg Foundation (E42/15), the Swedish Research Council (421-2013-1061, 2019-00244). MB is funded by an ERC Consolidator Grant (WELL-BEING; grant 771057). JBP is supported by the Medical Research Foundation 2018 Emerging Leaders 1st Prize in Adolescent Mental Health (MRF-160-0002-ELP-PINGA) and the European Research Council (ERC) under the European Union's Horizon 2020 research and innovation programme (grant agreement No. 863981). WDH is supported by Age UK (Disconnected Mind grant), and by a Career Development Award from the Medical Research Council (MRC) [MR/T030852/1] for the project titled "From genetic sequence to phenotypic consequence: genetic and environmental links between cognitive ability, socioeconomic position, and health". DME is supported by an NHMRC Senior Research Fellowship (GNT1137714). JLH is a NHMRC Senior Principal Research Fellow. SL is supported by a Victorian Cancer Agency Early Career Research Fellowhip (ECRF19020). MCS is a Senior Research Fellow of the National Health and Medicial Research Council of Australia. SEM and NGM are supported through NHMRC investigator grants (APP1172917 and APP1172990). BMB, BOÅ, HR, AFH and KH work in a research unit funded by Stiftelsen Kristian Gerhard Jebsen; Faculty of Medicine and Health Sciences, NTNU; The Liaison Committee for education, research and innovation in Central Norway; the Joint Research Committee between St. Olavs Hospital and the Faculty of Medicine and Health Sciences, NTNU. The genotyping in HUNT was financed by the National Institutes of Health; University of Michigan; the Research Council of Norway; the Liaison Committee for Education, Research and Innovation in Central Norway; and the Joint Research Committee between St Olavs hospital and the Faculty of Medicine and Health Sciences, NTNU. ON receives support from own institution and Norwegian Research Council (RCN) grant number 287347. AH was supported by the South-Eastern Norway Regional Health Authority (2020022 and 2021045) and the Research Council of Norway (274611, 288083 and 300668). EC was supported by the South-Eastern Norway Regional Health Authority (2021045) and the Research Council of Norway (274611). MCM is supported by the Leverhulme Trust, Leverhulme Centre for Demographic Science, ERC 835079. CH is supported by an MRC University Unit Programme Grant MC_UU_00007/10 (QTL in Health and Disease). JKH was supported by DA011015 for collection of the data used in this paper, and is currently supported by U01DA051018, R01DA042755, and R01AG046938. MCK is supported by National Institute of Mental Health grant R01 MH100141. RA and SO are supported by Riksbankens Jubileumsfond (P18-0782:1) and the Swedish Research Council (2019-00244). TwinsUK and MM are supported by the National Institute for Health Research (NIHR)-funded BioResource, Clinical Research Facility and Biomedical Research Centre based at Guy's and St Thomas' NHS Foundation Trust in partnership with King's College London. PC is funded by the European

Union (H2020 contract #733100). PRN received support from the European Research Council (AdG SELECTionPREDISPOSED #293574), the Bergen Research Foundation ("Utilizing the Mother and Child Cohort and the Medical Birth Registry for Better Health"), Stiftelsen Kristian Gerhard Jebsen (Translational Medical Center), the University of Bergen, the Research Council of Norway (FRIPRO grant #240413), the Western Norway Regional Health Authority (Strategic Fund "Personalized Medicine for Children and Adults"), the Novo Nordisk Foundation (grant #54741), and the Norwegian Diabetes Association. PM is funded by the Academy of Finland (#308247, # 345219), and the European Research Council under the European Union's Horizon 2020 research and innovation programme (grant agreement No 101019329). CAR is currently supported by NIH grants RF1AG058068, R01AG060470, R01AG059329, R01AG050595, and R01AG046938. SH is supported by Karolinska Institutet Strategic Research Program in Epidemiology and the Swedish Research Council (2019-01272).

Cohort descriptions

Australian Mammographic Density Twins and Sisters Study

Overview

Australian Mammographic Density Twins and Sisters Study (AMDTSS) is a twin and family study for studying mammographic density (Odefrey et al 2010). Female twins and their sisters without breast cancer were recruited between 2004 and 2009. Participants completed questionnaire surveys through telephone-administered interviews to be collected for self-reported weight, height, and other known and putative breast cancer risk factors. Blood samples were collected, couriered to the laboratory within 48 hours of collection, and were processed to generate dried blood spot Guthrie cards. The study was approved by the Human Research Ethics Committee of The University of Melbourne, and written informed consent was obtained from the participants. 1811 first-degree sisters (monozygotic twin pairs were excluded if there was no other sisters available) of 685 sibships were included in this study.

Genotyping, imputation and quality control

Genome-wide SNP data were genotyped using DNA extracted from blood samples and the Infinium OncoArray-500K Beadchip. Pre-imputation quality control included filtering SNPs to have call rate >=95%, MAF>=0.01 and HWE P>=10-7, and filtering samples to have call rate>0.95, sex consistency and heterozygosity rate Z<4.89. Imputation was conducted using the Michigan Imputation Server with HRC Release 1.1 as the reference panel. Post-imputation quality control included filtering SNPs to have MAF>=0.01, imputation quality info>=0.3, genotype rate >=95% and HWE P=10-7.

Acknowledgements

We would like to thank all participants of the AMDTSS. The AMDTSS was facilitated through access to Twins Research Australia, a national resource supported by a Centre of Research Excellence Grant (number 1079102) from the NHMRC. The AMDTSS was supported by NHMRC (grant numbers 1050561 and 1079102), Cancer Australia and National Breast Cancer Foundation (grant number 509307).

References

1. Odefrey, F., Stone, J., Gurrin, L. C., Byrnes, G. B., Apicella, C., Dite, G. S., . . . Southey, M. C. (2010). Common genetic variants associated with breast cancer and mammographic density measures that predict disease. Cancer Research, 70, 1449–1458.

The Center on Antisocial Drug Dependence (CADD)

Overview
The Center on Antisocial Drug Dependence (CADD) was established in 1997 to investigate the etiology of substance use disorders and related conditions. This study includes individuals from two cohorts within CADD: the longitudinal twin sample (LTS) and the community twin sample (CTS) (Rhea et al., 2013). Recruited in assistance with the Colorado Department of Health (CDH), the LTS sample consists of twins recruited at birth and tested annually. The CTS sample, in comparison, was recruited in collaboration with both CDH and the Colorado Department of Education to include twins ranging in age from 12-18 at the first point of assessment. In both samples, the closest-in-age sibling was included. All analyses were performed using Wave 2 of the LTS and CTS data.

Genotyping, imputation and quality control
Individuals were genotyped using the Affymetrix Axiom Precision Medicine Research Array (PMRA) Chip. Pre-imputation quality control included filtering SNPs to have call rate ≥ 95%, MAF ≥ 0.0001, HWE $p \geq$ 10-6, and filtering samples to have call rate > 0.95. Imputation was conducted using EAGLE (version 2.4). Post-imputation quality control included filtering SNPs to have MAF ≥ 0.0001, imputation quality INFO > 0.3, SNP call rate ≥ 95% and HWE $p \geq$ 10-6.

Analysis
Analyses were performed using the supplied scripts and using age, sex, and 20 principal components as covariates. The initial assessment of height and weight in these samples were in inches and pounds. These measures were subsequently converted to metric units to derive height (cm) and BMI (kg/m^2).

Acknowledgements
We would like to acknowledge and thank all the participants. This research was supported by the National Institute on Drug Abuse (Grant Numbers: DA038504 and DA011015), and utilized resources from the University of Colorado Boulder Research Computing Group, which is supported by the National Science Foundation (Award Nos. ACI-1532235 and ACI1532236), the University of Colorado Boulder, and Colorado State University.

References

1. Rhea, S.A., Gross, A. A., Haberstick, B. C., & Corley, R. P. (2006). Colorado Twin Registry. Twin Research and Human Genetics, 9(6), 941-949. PMID 17254434
2. Rhea, S.A., Gross, A.A., Haberstick, B.C., Corley, R.P. (2013). Colorado Twin Registry: An Update. Twin Research and Human Genetics, 16(1), 351-7.

China Kadoorie Biobank (CKB)

Overview

China Kadoorie Biobank is a prospective population-based cohort of 512,891 adults aged 30-79 years recruited from 10 geographically defined regions during 2004-2008 (1). The baseline survey collected questionnaire data, physical measurements, and blood samples, including measurement of blood glucose with recording of time since last meal. Five-yearly resurveys are undertaken among a 5%

randomly-selected sample of surviving participants, collecting the same information as at the baseline survey plus some additional measures. All participants are followed for cause-specific mortality and morbidity and for any hospital admission, through linkages with registries and health insurance databases. Local, national and international ethics approval was obtained and all participants provided written informed consent.

Genotyping, imputation and quality control

Genotyping was conducted using custom Affymetrix Axiom® arrays, with 100,706 unique samples (call rate >0.95, no sex mismatch, no XY aneuploidy, heterozygosity < mean+3SD) and 511,885 variants (call rate >0.98, HWE P>1E-06, batch/plate effect P<1E-06, MAF difference from 1000 genomes EAS <0.2) passing QC. Genotypes were phased using SHAPEIT3 v4.12 and imputed into the 1000 Genomes Phase 3 reference with IMPUTE4 v4.r265. After imputation, variants with MAF<0.005 or info<0.3 were excluded. Genotype probabilities were converted to hard-call genotypes in Plink using a cut-off of 0.499 (i.e. the most probable genotype).

Sib-pairs were initially identified as sample pairs with pi-hat>0.375, excluding parent-child pairs by confirming Z0 >0.05, Z1>0.5. Family structures were checked to ensure all family members were sibs, where necessary excluding individuals to remove putative ½- or ¾-sibling relationships. Sole monozygotic twins (i.e. with no other siblings in the dataset) were excluded.

Analysis

Analyses were performed using the supplied scripts, including as covariates: age at baseline; sex, recruitment region, and 12 principal components. Systolic blood pressure was adjusted for use of BP-lowering medication by adding 15 mmHg. Alcohol consumption was derived from questionnaire responses as previously described (2).

Acknowledgements

China Kadoorie Biobank was supported as follows: Baseline survey and first re-survey: Hong Kong Kadoorie Charitable Foundation; long-term follow-up: UK Wellcome Trust (212946/Z/18/Z, 202922/Z/16/Z, 104085/Z/14/Z, 088158/Z/09/Z), National Natural Science Foundation of China (91843302), and National Key Research and Development Program of China (2016YFC 0900500, 0900501, 0900504, 1303904). DNA extraction and genotyping: GlaxoSmithKline, UK Medical Research Council (MC_PC_13049, MC-PC-14135). The UK Medical Research Council (MC_UU_00017/1,MC_UU_12026/2 MC_U137686851), Cancer Research UK (C16077/A29186; C500/A16896) and the British Heart Foundation (CH/1996001/9454), provide core funding to the Clinical Trial Service Unit and Epidemiological Studies Unit at Oxford University for the project. CKB acknowledges the study participants and members of the survey teams in each of the 10 regional centres, and the project development and management teams based at Beijing, Oxford and the 10 regional centres.

References

1. Chen Z *et al.* China Kadoorie Biobank of 0.5 million people: survey methods, baseline characteristics and long-term follow-up. *Int J Epidemiol* **40**, 1652-1666 (2011).
2. Millwood IY *et al.* Conventional and genetic evidence on the association between alcohol and vascular disease in 500 000 adults in China: a prospective cohort study. *The Lancet* 393, 1831-1842 (2019).

Danish Twin Registry (DTR)

Overview

This study included 586 dizygotic twin pairs recruited by the Danish Twin Registry (DTR) as part of the study of Middle-Aged Danish Twins (MADT) and the Longitudinal Study of Aging Danish Twins (LSADT). Briefly, MADT was initiated in 1998 and includes 4,314 twins randomly chosen from the birth years 1931-1952. Surviving participants were revisited from 2008 to 2011 (1), where the blood samples used in this study were collected. LSADT was initiated in 1995 and includes twins aged 70 years and older. Follow-up assessments were conducted every second year through 2005 (1). The individuals included in the present study all participated in the 1997 assessment, where blood samples were collected from same sex twin pairs.

For MADT study participants, data on height, BMI, education, depressive symptoms, age at first birth, and physical activity were collected as part of the 1998 assessment, whereas data on cognition, smoking, alcohol consumption, subjective wellbeing, neuroticism, and number of biological children were collected as part of the 2008-2011 follow-up survey. All data for the LSADT study participants were collected as part of the 1997 assessment.

Written informed consents were obtained from all participants. Collection and use of biological material and survey information were approved by the Regional Scientific Ethical Committees for Southern Denmark, and the study was approved by the Danish Data Protection Agency.

Genotyping, imputation and quality control

DNA was extracted from whole blood using a manual (2) or a semi-automatic (Autopure, Qiagen, Hilden, Germany) salting out method. Samples were genotyped using the Illumina Infinium PsychArray (Illumina San Diego, CA, USA). Pre-imputation quality control included filtering SNPs on genotype call rate <98%, HWE $P<10^{-6}$, and MAF = 0, and individuals on sample call rate <99%, relatedness and gender mismatch. Pre-phasing and imputation to the 1000 Genomes phase 3 reference panel was performed using IMPUTE2 version 2.3.2 (3). After imputation, genotype probabilities were converted to hard-called genotypes in Plink using a cut-off of 90%, meaning that only genotypes with a probability of more than 90% were called. Variants with no genotype probabilities above 90% were set to missing. Post-imputation quality control included filtering variants on MAF < 0.01 and INFO < 0.30, and the removal of CNVs and duplicate variants.

Analysis

Analyses were performed using the supplied scripts including age at phenotyping, gender and 20 principal components as covariates.

Acknowledgements

This study was supported by The National Program for Research Infrastructure 2007 (grant no. 09-063256), the Danish Agency for Science Technology and Innovation, and the US National Institute of Health (P01 AG08761). Genotyping was supported by NIH R01 AG037985 (Pedersen). Genotyping was conducted by the SNP&SEQ Technology Platform, Science for Life Laboratory, Uppsala, Sweden (http://snpseq.medsci.uu.se/genotyping/snp-services/).

References

1. Pedersen DA, Larsen LA, Nygaard M, Mengel-From J, McGue M, Dalgård C, Skytthe A, Kyvik KO, Hvidberg L, Hjelmborg JvB, Holm NV, Christensen K. The Danish Twin Registry: An Updated Overview. Twin Research and Human Genetics, 1-9. DOI: 10.1017/thg.2019.72.

2. Miller SA, Dykes DD and Polesky HF. A simple salting out procedure for extracting DNA from human nucleated cells. Nucleic acids research. 1988; 16(3):1215.

3. Howie BN, Donnelly P, Marchini J. A flexible and accurate genotype imputation method for the next generation of genome-wide association studies. PLoS Genetics. 2009; 5(6): e1000529.

DiscovEHR Study

Overview

Geisinger is the largest health care provider in central Pennsylvania with ~2 million patients. The DiscovEHR study, a subset of Geisinger's MyCode Community Health Initiative cohort, with electronic medical records linked to genetic data (http://www.discovehrshare.com/). Geisinger patients enrolled in MyCode consent to broad research use of their samples. At the time of analysis, DiscovEHR included 92,476 consented individuals 92,476 with genetic data and with a median of 14 years of follow-up with EHR-derived clinical data.

For the purposes genetically-informed European-ancestry individuals were used for all analyses. For BMI and height adults aged 20-70 years. For height, unrealistic and outlier values were excluded (<44 inches, > 84 inches, and +/- 5 SD from mean). Height measurements were subsequently converted to metric (cm). Unrealistic and outlier weights <52 lbs, >650 lbs, and +/- 5 SD from mean were excluded. Additionally, weights during or within six months of pregnancy or following weight

loss surgery were excluded. Weight in lbs was subsequently converted to metric units (kg). For individuals with repeated weight measurements. Cleaned height and weight were used to calculate BMI (kg/m^2) following data cleaning and conversion to metric units. For individuals with more than one measured height, median height in cm was used in all analyses. For repeated BMI, maximum BMI was used on all GWAS analyses. For SBP measures were extracted from clinical labs for adults aged 18 to 89 years. SBP measures were excluded on or following a diagnosis of chronic kidney disease (ICD-9 585), congestive heart failure (ICD-9 428), secondary hypertension (ICD-9 405). Additionally, SBP measurements were excluded if patient reported severe pain during encounter. SBP measurements were adjusted by adding 15 mmHg to measurement if patient was currently taking blood pressure lowering medication. For patients with multiple measures, median SBP was used in GWAS analyses.

Genotyping, imputation and quality control

Genotyping and quality control of DiscovEHR data have been previously described (Staples et al. 2018). Briefly, of the 92,476 with array-based genotyping, 67% were genotyped using the Illumina HumanOmniExpressExome (HOEE) genotyping platform with remainder typed on the Illumina Global Screening Array (GSA). These data are processed with Illumina's GenomeStudio, imputed to the Haplotype Reference Consortium data and merged, resulting in 7.6 million variants. All array based data are QC'd with standard quality control procedures before association testing (Turner et al. 2011). Following imputation, variant dosages were filtered on INFO>0.3 and MAF>0.01, following the current study recommendations.

Analysis

All GWAS analyses were adjusted for sex, age (concurrent to phenotype measurement as described above, i.e. age at maximum BMI), array platform, and the first 20 PCs calculated from whole exome sequences.

References

1. Turner, S. *et al.* Quality control procedures for genome-wide association studies. *Curr Protoc Hum Genet* **Chapter 1**, Unit1 19 (2011).

2. Staples J, Maxwell EK, Gosalia N, et al. Profiling and Leveraging Relatedness in a Precision Medicine Cohort of 92,455 Exomes. *Am J Hum Genet*. 2018;102(5):874-889. doi:10.1016/j.ajhg.2018.03.012

Finnish Twin Cohort

Overview

The Finnish Twin Cohort consists of three longitudinal twin-family cohorts (1,2,3). The oldest cohort was initiated in 1975 and consists of twin pairs born before 1958 (3), while the FinnTwin12 (twins born 1983-1987) and FinnTwin16 (twins born 1975-1979) started in the 1990s (1,2). Each cohort has participated in 4 to 5 waves of data collection by mailed or online questionnaires, while some twin pairs and siblings have been invited to in-person studies, or telephone interviews with collection of DNA from venous blood samples or saliva. Phenotypes were compiled from these data sources such that the data from a twin pair was always at the same time point. The individual data collections have been approved by the respective data authorities, ethical committees and IRBs as documented in our reviews (1,2,3). Most sibling pairs included in the analyses are dizygotic twins, but some are also non-twin siblings.

Genotyping, imputation and quality control

Genotyping was done using Illumina Human610-Quad v1.0 B and Human670-QuadCustom v1.0 arrays at the Wellcome Trust Sanger Institute (Cambridge UK), Illumina HumanCoreExome- (12 v1.0 A, 12 v1.1 A, 24 v1.0 A, 24 v1.1 A, 24 v1.2 A) arrays at the Broad Institute of MIT and Harvard (MA, USA), Wellcome Trust Sanger Institute (Cambridge, UK), University of Chicago Genomics Facility (Chicago IL, USA) and Institute for Molecular Medicine Finland (Helsinki, Finland) and with Affymetrix FinnGen Axiom array at Thermo Fisher Scientific (Santa Clara CA, USA). The algorithm for genotype calling were Illumina's GenCall for all HumanCoreExome chip genotypes, Illuminus for 610k & 670k chip genotypes and AxiomGT1 for Affymetrix chip genotypes. On Illumina arrays where genotypes were called to Illumina's TOP strand, strand were flipped to forward strand using strand files generated by Will Rayner (https://www.well.ox.ac.uk/~wrayner/strand/). The genome build of all genotypes were set to GRCh37/hg19. In case where genotypes were called to NCBI36/hg18 or GRCh38/hg38, genome positions were lifted to GRCh37/hg19 using University of California Santa Cruz LiftOver program [4] with appropriate chain file. Genotype quality control were done in three batches (batch1: 610k+670k, batch2: HumanCoreExome and batch3: Affymetrix chip genotypes). Variants with call rate below 97.5% (batch1 and batch3) or 95% (batch2), samples with call rate below 98% (batch1) or 95% (batch2 and batch3), variants with minor allele frequency below 1% with Hardy-Weinberg Equilibrium p-value lower than 1e-06 were removed. In addition, amples from all batches with heterozygosity test method-of-moments F coefficient estimate value below -0.03 or higher than 0.05 (batch1 and batch2) or ±4SD from the mean (batch3) were removed along with the samples which failed sex check or were among the multi-dimensional scaling principal component analysis outliers. Total amount of genotyped autosomal variants after quality control were 475526 (batch1), 239894 (batch2) and 388673 (batch3) with following number of samples remaining for imputation: 2617 (batch1), 5328 (batch2) and 8218 (batch3). We then performed pre-phasing using Eagle v2.3 [5] and imputation with Minimac3 v2.0.1 using University of Michigan Imputation Server [6]. Genotypes of all batches were imputed to Haplotype Reference Consortium release 1.1 reference panel [7]. The final study sample were extracted from the data where all batches of imputed data were merged. As post-imputation quality control variants with MAF below 1% and imputation quality score below 0.5 were removed.

Analysis

Analyses were performed with the scripts supplied with this project using age, sex and 20 principal components as covariates.

References

1: Kaidesoja M, Aaltonen S, Bogl LH, Heikkilä K, Kaartinen S, Kujala UM, Kärkkäinen U, Masip G, Mustelin L, Palviainen T, Pietiläinen KH, Rottensteiner M, Sipilä PN, Rose RJ, Keski-Rahkonen A, Kaprio J. FinnTwin16: A Longitudinal Study from Age 16 of a Population-Based Finnish Twin Cohort. Twin Res Hum Genet. 2019 ;22(6):530-539. doi: 10.1017/thg.2019.106. . PMID: 31796134.

2: Rose RJ, Salvatore JE, Aaltonen S, Barr PB, Bogl LH, Byers HA, Heikkilä K, Korhonen T, Latvala A, Palviainen T, Ranjit A, Whipp AM, Pulkkinen L, Dick DM, Kaprio J. FinnTwin12 Cohort: An Updated Review. Twin Res Hum Genet. 2019;22(5):302-311. doi: 10.1017/thg.2019.83. . PMID: 31640839;

3: Kaprio J, Bollepalli S, Buchwald J, Iso-Markku P, Korhonen T, Kovanen V, Kujala U, Laakkonen EK, Latvala A, Leskinen T, Lindgren N, Ollikainen M, Piirtola M, Rantanen T, Rinne J, Rose RJ, Sillanpää E, Silventoinen K, Sipilä S, Viljanen A, Vuoksimaa E, Waller K. The Older Finnish Twin Cohort - 45 Years of Follow-up. Twin Res Hum Genet. 2019;22(4):240-254. doi: 10.1017/thg.2019.54.. PMID: 31462340.

4. Hinrichs et al. The UCSC Genome Browser Database: update 2006. Nucleic Acid Research 2006. PMID: 16381938

5. Loh et al. Reference-based phasing using the Haplotype Reference Consortium panel. Nature Genetics 2016. PMID: 27694958

6. Das et al. Next-generation genotype imputation service and methods. Nature Genetics 2016. PMID: 27571263

7. the Haplotype Reference Consortium. A reference panel of 64,976 haplotypes for genotype imputation. Nature Genetics 2016. PMID: 27548312

<u>Generation Scotland</u>

Overview

The Generation Scotland: Scottish Family Health Study (GS:SFHS) is a family-based population cohort with DNA, biological samples, socio-demographic, psychological and clinical data from approximately 24,000 adult volunteers across Scotland. Although data collection was cross-sectional, GS:SFHS became a prospective cohort due to of the ability to link to routine Electronic Health Record (EHR) data. Over 20,000 participants were selected for genotyping using a large genome-wide array.

Genotyping, imputation and quality control

GS_SFHS waas genotyped using either the HumanOmniExpressExome8v1-2_or the HumanOmniExpressExome-8v1_A . Pre-imputation quality control included filtering SNPs on genotype call rate <98%, HWE P<10^{-6}, and individuals on sample call rate <98%. MAF was >0.01 for OMNI markers and >0.0001 for Exome Chip markers. 602450 SNPS were phased using Shapeit v2.r873 + duohmm and imputed imputed using the Haplotype Research Consortium (HRC.r1-1) dataset.

Analysis

Analyses were performed using the supplied scripts including age at phenotyping, gender and 20 principal components as covariates.

Acknowledgements

Generation Scotland received core support from the Chief Scientist Office of the Scottish Government Health Directorates [CZD/16/6] and the Scottish Funding Council [HR03006] and is currently supported by the Wellcome Trust [216767/Z/19/Z]. Genotyping of the GS:SFHS samples was carried out by the Genetics Core Laboratory at the Edinburgh Clinical Research Facility, University of Edinburgh, Scotland and was funded by the Medical Research Council UK and the Wellcome Trust (Wellcome Trust Strategic Award "STratifying Resilience and Depression Longitudinally" (STRADL) Reference 104036/Z/14/Z. C.H is supported by an MRC University Unit Programme Grant MC_UU_00007/10 (QTL in Health and Disease). We are grateful to all the families who took part, the general practitioners and the Scottish School of Primary Care for their help in recruiting them, and the whole Generation Scotland team, which includes interviewers, computer and laboratory technicians, clerical workers, research scientists, volunteers, managers, receptionists, healthcare assistants and nurses.

References

1. Smith BH, Campbell A, Linksted P, Fitzpatrick B, Jackson C, Kerr SM, Deary IJ, Macintyre DJ, Campbell H, McGilchrist M, Hocking LJ, Wisely L, Ford I, Lindsay RS, Morton R, Palmer CN, Dominiczak AF, Porteous DJ, Morris AD. Cohort profile: Generation Scotland: Scottish Family Health Study (GS:SFHS). The study, its participants and their potential for genetic research on health and illness. Int J Epidemiol. 2012;42(3):689–700. doi: 10.1093/ije/dys084.
2. Nagy R, Boutin TS, Marten J, Huffman JE, Kerr SM, Campbell A, Evenden L, Gibson J, Amador C, Howard DM, Navarro P, Morris A, Deary IJ, Hocking LJ, Padmanabhan S, Smith BH, Joshi P, Wilson JF, Hastie ND, Wright AF, McIntosh AM, Porteous DJ, Haley CS, Vitart V, Hayward C. Exploration of haplotype research consortium imputation for genome-wide association studies in 20,032 Generation Scotland participants. Genome Med. 2017 Mar 7;9(1):23. doi: 10.1186/s13073-017-0414-4. PMID: 28270201

Genetic Epidemiology Network of Arteriopathy (GENOA)

Overview

The Genetic Epidemiology Network of Arteriopathy (GENOA) study is a community-based study of hypertensive sibships that was designed to investigate the genetics of hypertension and target organ damage in African Americans from Jackson, Mississippi and non-Hispanic whites from Rochester, Minnesota (Daniels, 2004). In the initial phase of GENOA (Phase I: 1996-2001), all members of sibships containing ≥ 2 individuals with essential hypertension clinically diagnosed before age 60 were invited to participate, including both hypertensive and normotensive siblings. Exclusion criteria of the GENOA study were secondary hypertension, alcoholism or drug abuse, pregnancy, insulin-dependent diabetes mellitus, or active malignancy. Eighty percent of African Americans (1,482 subjects) and 75% of non-Hispanic whites (1,213 subjects) from the initial study population returned for the second examination (Phase II: 2001-2005). Study visits were made in the morning after an overnight fast of at least eight hours. Demographic information, medical history, clinical characteristics, lifestyle factors, and blood samples were collected in each phase. Written informed consent was obtained from all subjects and approval was granted by participating institutional review boards. Phenotypes and covariates were first processed in SAS 9.4.

All phenotypes were from GENOA Phase I except the cognitive phenotypes which were taken from an ancillary study conducted approximately 1 year after Phase II. Height in centimetres, BMI, and waist-to-hip ratio were calculated from measurements taken at in-person examinations. WHR was then adjusted for BMI with the residuals from linear regression used for this analysis. Education was the number of years of schooling. Sitting systolic blood pressure (SBP) (mmHg) was measured three times with a random zero sphygmomanometer. The average of the last two measurements was used in this study and adjusted by 10 units if an antihypertensive medication was taken. The general cognitive function score was the first unrotated principal component constructed by Principal Component Analysis (PCA) using the following 5 test measures: 1) the REY Auditory Verbal Learning Delayed Recall Test, 2) the Digital Symbol Substitution Test, 3) the FAS Word Fluency Test, 4) the Stroop Color Word Test, and 5) Part A of the Trail Making Test. Smoking phenotypes were assessed from self-report and include average cigarettes per day, ever smoker (more than 100 lifetime cigarettes), and age of initiation (>30 set to missing). Alcohol consumption was the average number of alcoholic units consumed per week as reported by the participant, aggregated across all types of alcohol, and outliers >5SD from the mean were removed. CRP and cholesterol measures were measured from blood. CRP was natural log transformed and standardized to mean 0 and SD 1. LDL was calculated as (total cholesterol) - HDL - (triglycerides/5), and all cholesterol measures were standardized to mean 0 and SD 1. eGFR was calculated from the CKD-EPI equation. Physical activity was defined as more than 20 minutes per week of moderate/vigorous activity.

Genotyping, imputation and quality control

All GENOA participants were genotyped on the Affymetrix Genome-Wide Human SNP Array 6.0 or the Illumina Human 1M-Duo BeadChip. Samples were removed if they had a missing call rate ≥0.05 or were an outlier ≥6 standard deviations from the mean of the first 10 genome-wide principal components from genotype data. SNPs were filtered to include those with MAF >0.01 and r 2 (INFO) >0.3. Imputation was done on the Michigan Imputation server (https://imputationserver.sph.umich.edu/index.html#!pages/home) using SHAPEIT and minimac3.

Genotypes were imputed to the Haplotype Reference Consortium (HRC version r1.1) reference panel.

Acknowledgements

Support for the Genetic Epidemiology Network of Arteriopathy (GENOA) was provided by the National Heart, Lung and Blood Institute (U01 HL054464, U01 HL054457, U01 HL054481, R01 HL087660, R01 HL085571, R01 HL119443) and the National Institute of Neurological Disorders and Stroke (R01 NS041558). Genotyping was performed at the University of Texas Health Sciences Center. We would like to thank the families that participated in the GENOA study.

References

Daniels PR, et al. (2004) Familial aggregation of hypertension treatment and control in the Genetic Epidemiology Network of Arteriopathy (GENOA) study. Am J Med. 116(10):676-81.

<u>HUNT</u>

Overview

The Trøndelag Health Study (The HUNT Study) is a longitudinal population-based health study conducted in the county of Trøndelag, Norway. Data and samples have been collected through four surveys (HUNT1 [1984-1986], HUNT2 [1995-1997], HUNT3 [2006-2008] and HUNT4 [2017-2019]) (1). At each time point, the entire adult population (≥ 20 years) was invited to participate by completing questionnaires, attending clinical examinations and interviews.

Genotyping, imputation and quality control

In total, DNA from 71,860 HUNT participants was genotyped using one of three Illumina HumanCoreExome arrays (HumanCoreExome12 v1.0, HumanCoreExome12 v1.1 and UM HUNT Biobank v1.0). Quality control was performed at the marker and sample level. Samples that failed to reach a 99% call rate, had contamination > 2.5% as estimated with BAF Regress (2), large chromosomal copy number variants, lower call rate of a technical duplicate pair and twins, gonosomal constellations other than XX and XY, or whose inferred sex contradicted the reported gender, were excluded. Samples that passed quality control were analyzed in a second round of genotype calling following the Genome Studio quality control protocol described elsewhere (3). Variants were excluded if their probe sequences could not be perfectly mapped to the reference genome, cluster separation < 0.3, Gentrain score < 0.15, deviations from Hardy Weinberg equilibrium in unrelated samples of European ancestry with p-value < 0.0001, call rate < 99%, or another assay with higher call rate genotyped the same variant. Imputation was performed using Minimac3 (v2.0.1, http://genome.sph.umich.edu/wiki/Minimac3) (4) and a merged reference panel that was constructed by combining the Haplotype Reference consortium release 1.1 (HRC v1.1) and a local reference panel based on 2,202 whole-genome sequenced HUNT study participants. SNPs were filtered to include those with MAF > 0.01 and Rsq > 0.3.

Analysis

Analyses were performed with the scripts supplied with this project using age, sex, 20 principal components and genotyping batch as covariates.

Acknowledgements

The HUNT Study is a collaboration between HUNT Research Centre, (Faculty of Medicine and Health Sciences, NTNU, Norwegian University of Science and Technology), Trøndelag County Council, the Central Norway Regional Health Authority, and the Norwegian Institute of Public Health. The genotyping in HUNT was financed by the National Institutes of Health (NIH); University of Michigan; The Research Council of Norway; The Liaison Committee for Education, Research and Innovation in Central Norway; and the Joint Research Committee between St. Olavs hospital and the Faculty of Medicine and Health Sciences, NTNU. The genotype quality control and imputation has been conducted by the K.G. Jebsen Center for Genetic Epidemiology, Department of Public Health and Nursing, Faculty of Medicine and Health Sciences, NTNU, Norwegian University of Science and Technology.

References

1. Krokstad, S. et al. Cohort profile: The HUNT study, Norway. Int. J. Epidemiol. 42, 968–977 (2013).

2. Jun, G. et al. Detecting and estimating contamination of human DNA samples in sequencing and array-based genotype data. Am. J. Hum. Genet. 91, 839–848 (2012).

3. Guo, Y. et al. Illumina human exome genotyping array clustering and quality control. Nat. Protoc. 9, 2643–2662 (2014).

4. Das, S. et al. Next-generation genotype imputation service and methods. Nat. Genet. 48, 1284–1287 (2016).

Minnesota Center for Twin & Family Research (MCTFR)

Overview

The MCTFR GWAS sample (1) represents participants from three longitudinal studies: The Minnesota Twin Family Study (MTFS; 2), the Sibling Interaction and Behavior Study (SIBS, 3), and the Enrichment Study (ES; 4). These studies share similar assessment protocols and a common sampling unit, a four-member family consisting of sibling pairs and their rearing parents. Offspring in all three samples were initially assessed in adolescence and followed into at least early adulthood. Intake assessment included a total of 9,827 individuals (5,001 offspring), with quality control (QC) filters producing a final GWAS sample of 7,689 individuals representing 2,390 families. Of these, 830 individuals from 415 dizygotic or regular sibships were of European ancestry and had phenotypes for

one or more of the following: Height, BMI, IQ, educational attainment, income, ever-smoker, drinks per week, risk taking, neuroticism, systolic blood pressure, and subjective wellbeing.

Genotyping, imputation and quality control

Genome-wide genotyping was carried out using the Illumina Human660W-Quad array (Illumina, Inc., San Diego, CA) as per the manufacturer's protocol (5). This Infinium HD Beadchip required 200ng DNA per sample and contains 657,366 variants. In addition to standard QC filters, GenCall scores, which are metrics of genotype reliability generated by the BeadStudio software (Illumina Corporation, San Diego, California), were used to assess sample quality (6). For an additional check on quality control, each sample was genotyped on a custom 96-plex panel using IlluminaVeraCode chemistry (7), which contains SNPs present on the Human660W-Quad. SNP markers were subjected to nine standard QC filters, including sex mismatch, call rate < 99%, MAF < 1%, and HWE $p < 10^{-7}$. A total of 5.7% of the markers attempted failed one or more filters, leaving 527,829 markers that passed all QC filters. EIGENSTRAT (8) was used to extract the first 10 principal components and to aid in identification of the European ancestry cluster. Untyped genotypes were imputed to the Haplotype Reference Consortium through the Michigan Imputation Server, using Minimac3. Following the study recommendations, genotypes with MAF < 0.01 and imputation quality score < 0.3 were removed, resulting in 7,720,114 variants.

Analysis

Analyses were performed with the scripts supplied with this project using age, sex and 10 principal components as covariates.

Acknowledgements

MCTFR recruitment, assessment and genotyping was supported in part by USPHS Grants from the National Institute on Alcohol Abuse and Alcoholism (AA09367 and AA11886), the National Institute on Drug Abuse (DA05147, DA13240, and DA024417), and the National Institute on Mental Health (MH066140).

References

1. Miller, M. B., Basu, S., Cunningham, J., Eskin, E., Malone, S. M., Oetting, W. S., Schork, N., Sul, J. H., Iacono, W. G., & McGue, M. (2012). The Minnesota Center for Twin and Family Research genome-wide association study. Twin Research and Human Genetics, 15(6), 767–774.

2. Iacono, W. G., Carlson, S. R., Taylor, J., Elkins, I. J., & McGue, M. (1999). Behavioral disinhibition and the development of substance use disorders: Findings from the Minnesota Twin Family Study. *Development and Psychopathology, 11*, 869–900.

3. McGue, M., Keyes, M., Sharma, A., Elkins, I., Legrand, L., Johnson, W., & Iacono, W. G. (2007). The environments of adopted and non-adopted youth: Evidence on range restriction from the Sibling Interaction and Behavior Study (SIBS). *Behavior Genetics, 37*(3), 449–462.

4. Keyes, M. A., Malone, S. M., Elkins, I. J., Legrand, L. N., McGue, M., & Iacono, W. G. (2009). The Enrichment Study of the Minnesota Twin Family Study: Increasing the yield of twin

families at high risk for externalizing psychopathology. *Twin Research and Human Genetics, 12*(5), 489– 501.

5. Li,Y.F., Sheu,C.C., Ye,Y.Q., de Andrade, M.,Wang, L., Chang, S. C., ... Yang, P. (2010). Genetic variants and risk of lung cancer in never smokers: A genome-wide association study. *Lancet Oncology, 11*(4), 321–330.

6. Cunningham, J. M., Sellers, T. A., Schildkraut, J. M., Fredericksen, Z. S., Vierkant, R. A., Kelemen, L. E., ... Goode, E. L. (2008). Performance of amplified DNA in an Illumina GoldenGate BeadArray assay. *Cancer Epidemiology Biomarkers & Prevention, 17*(7), 1781–1789.

7. Lin, C. H., Yeakley, J. M., McDaniel, T. K., & Shen, R. (2009). Medium- to high-throughput SNP genotyping using Vera-Code microbeads. *Methods in Molecular Biology, 496*, 129–142.

8. Price, A. L., Patterson, N. J., Plenge, R. M., Weinblatt, M. E., Shadick, N. A., & Reich, D. (2006). Principal components analysis corrects for stratification in genome-wide associa- tion studies. Nature Genetics, 38(8), 904–909.

9. Browning, B. L., &Browning, S. R. (2009). A unified approach to genotype imputation and haplotype-phase inference for large data sets of trios and unrelated individuals. American Journal of Human Genetics, 84(2), 210–223.

10. Li, Y.,Willer, C. J., Ding, J., Scheet, P.,&Abecasis, G. R. (2010). MaCH:Using sequence and genotype data to estimate hap- lotypes and unobserved genotypes. Genetic Epidemiology, 34(8), 816–834.

Norwegian Mother, Father and Child Cohort Study (MoBa)

Overview

The Norwegian Mother, Father and Child Cohort Study (MoBa) is a population-based pregnancy cohort study conducted by the Norwegian Institute of Public Health. Participants were recruited from all over Norway from 1999-2008. The women consented to participation in 41% of the pregnancies. The cohort now includes 114,500 children, 95,200 mothers and 75,200 fathers. The current study is based on version 12 of the quality-assured data files released for research on January 2019. The establishment of MoBa and initial data collection was based on a license from the Norwegian Data Protection Agency and approval from The Regional Committees for Medical and Health Research Ethics. The MoBa cohort is now based on regulations related to the Norwegian Health Registry Act. MoBa was linked to the Medical Birth Registry (MBRN), a national health registry containing information about all births in Norway. The current study was approved by The Regional Committees for Medical and Health Research Ethics (2016/1702).

Genotyping, imputation and quality control

The data were derived from the MoBa Genetics genotype data release 1.0 (n samples=98,110), based on genotype data from four research projects. 33,199 individuals in the NORMENT project were genotyped at deCODE genetics, Reykjavik Iceland (Illumina HumanOmniExpress-24v1.0,

Illumina InfiniumOmniExpress-24v1.2, & Illumina Global Screening Array MD v.1.0 + 50k custom OmniExpress overlap content array), 26,990 were genotyped in the Njølstad project at ERASMUS MC, Rotterdam, Netherlands (Illumina Global Screening Array MD v.1.0 array), and 5,410 from the ADHD project (TED) were genotyped at deCODE genetics (Illumina InfiniumOmniExpress-24v1.2), and 32,538 were sampled in the HARVEST sample at Genomics Core Facility, Trondheim, Norway (llumina HumanCoreExome12v1.1 & Illumina HumanCoreExome24v1.0). Pre-imputation QC, phasing and imputation are described here and here

Post-imputation data quality control
Individuals with sex-mismatch (derived by comparing genetic sex and reported sex) or individuals with sex-chromosome aneuploidy or those that were not linkable with the phenotypic data were excluded from the analysis (n=508). We checked the dataset for Mendelian errors using PLINK's --mendel command with thresholds of 1% and 5% for the trio and variant error rate, respectively. This excluded 129 individuals and 1,293 variants. On average these variants were less precisely imputed, mean INFO=0.88 versus mean INFO=0.97 for the rest of the SNPs.

3,061 individuals were genotyped twice, and 52 individuals were genotyped 3 times. We checked the concordance of SNPs within pairs of duplicated samples, and 2 samples, that were indicated as duplicates by the MoBa data, had very low concordance (pi_hat<0.03), and were excluded. The remaining individuals had high concordance (pi_hat>0.8). Note there were some 2,474 pairs of samples that had concordance between 0.74 and 0.98. These individuals are almost certainly the same individuals and are unlikely to represent sample contamination (this would have been picked up at the genotype calling stage). Therefore, this heterogeneity is likely because samples for the same individuals are combined from different chips which have not been imputed to the same standard. We excluded 140,767 SNPs which were discordant for more than 5% of duplicated samples. One individual from each pair of duplicates was then dropped at random with a seed.

Ancestry

We restricted the sample to individuals of 'European' ancestry using the first 2 principal components of the MoBa data. The principal components were calculated by merging the MoBa data with the 1000g reference panel and projecting the 1000g PCs onto the MoBa data. We then compared the PC values in the MoBa samples versus each of the populations included in the reference panel. We excluded samples if they had values of PC1 and PC2 that were within the range of the non-European samples in the 1000g reference panel, leading to the exclusion of 668 non-European samples.

Degree of relatedness:

Estimated kinship coefficients using the KING with age to the nearest year included as a covariate. identified 86,175 pairs of known related individuals from the pedigree and a further 10,769 unknown related individuals.

Parental relationships were updated based on KING results. Before running KING, we restricted it to an independent set of high frequency SNPs (MAF>0.10, window=3000kb and LD R2 >0.9). KING estimates the relationships of all the individuals in the datasets and reconstructs families. The output is a list of family and within family IDs. KING updated family IDs for 24,022 individuals, and parental relationships for 21,361 individuals. Samples were flagged for exclusion if newly assigned relationships appeared to be errors (375 samples). This included where: parents were less than 15 years older than children, both parents in a family were of the same sex, individuals were identified

as MZ twins but linked to different pregnancies, and siblings had different parents or an age gap of more than 25 years. We also flagged for exclusion parent-offspring pairs where the mother or father as identified by KING was different to the genotyped individual specified in the pedigree. This category will include both samples where the sampled partner is not the biological father, and potentially sample mix-ups.

Independently within the samples of parents and offspring we used GCTA to select an unrelated subsample using a threshold of IBS < 5% after pruning to an independent set of HAPMAP3 SNPs.

Principal components

The first 20 principal components were calculated independently for offspring and parents on a subset of the data limited to variants in HAPMAP3, that were pruned for independence using PLINK. We constructed two sets of principal components, the first used the MoBa data and accounts for structure within the data. The second set of principal components was generated using the 1000 genomes reference panel.

Analysis

Siblings were identified in the parents' generation using KING. We used the provided pipelines to analyse the data, with minor changes for the local infrastructure. We adjusted for sex, age, genotyping array, batch and 20 principal components.

The consent given by the participants does not open for storage of data on an individual level in repositories or journals. Researchers who want access to data sets for replication should submit an application to datatilgang@fhi.no. Access to data sets requires approval from The Regional Committee for Medical and Health Research Ethics in Norway and an agreement with MoBa.

Acknowledgements

This study includes data from the Norwegian Mother, Father and Child Cohort Study (MoBa) conducted by the Norwegian Institute of Public Health. The Norwegian Mother, Father and Child Cohort Study is supported by the Norwegian Ministry of Health and Care Services and the Ministry of Education and Research. We are grateful to all the participating families in Norway who take part in this on-going cohort study. We thank the Norwegian Institute of Public Health (NIPH) for generating high-quality genomic data. This research is part of the HARVEST collaboration, supported by the Research Council of Norway (#229624). We also thank the NORMENT Centre for providing genotype data, funded by the Research Council of Norway (#223273, 248778, 262656, 248980, 273291), South East Norway Health Authority and KG Jebsen Stiftelsen. We further thank the Center for Diabetes Research, the University of Bergen for providing genotype data and performing quality control and imputation of the data funded by the ERC AdG project SELECTionPREDISPOSED, Stiftelsen Kristian Gerhard Jebsen, Trond Mohn Foundation, the Research Council of Norway, the Novo Nordisk Foundation, the University of Bergen, and the Western Norway health Authorities (Helse Vest).

References

Magnus P, Birke C, Vejrup K, Haugan A, Alsaker E, Daltveit AK, Handal M, Haugen M, Hoiseth G, Knudsen GP, Paltiel L, Schreuder P; Tambs K, Vold L, Stoltenberg C. Cohort Profile Update: The Norwegian Mother and Child Cohort Study (MoBa). Int J Epidemiol 2016; 45:382-8.

Liv Paltiel, Anita Haugan, Trine Skjerden, Kari Harbak, Siri Bækken, Nina Kristin Stensrud, Gun Peggy Knudsen and Per Magnus. The biobank of the Norwegian Mother and Child Cohort Study – present status Nor J Epidemiol 2014; 24 (1-2): 29-35.

Helgeland, Øyvind, Marc Vaudel, Pol Sole-Navais, Christopher Flatley, Julius Juodakis, Jonas Bacelis, Ingvild L. Koløen, Gun Peggy Knudsen, Bente B. Johansson, Per Magnus, Ted Reichborn Kjennerud, Petur B. Juliusson, Camilla Stoltenberg, Oddgeir L. Holmen, Ole A. Andreassen, Bo Jacobsson, Pål R. Njølstad, and Stefan Johansson. 2021. Characterization of the Genetic Architecture of BMI in Infancy and Early Childhood Reveals Age-Specific Effects and Implicates Pathways Involved in Mendelian Obesity. MedRxiv 2021.05.04.21256508. doi: 10.1101/2021.05.04.21256508.

Netherlands Twin Register (NTR)

Overview

The Netherlands Twin Register (NTR, Ligthart et al. 2019) is an ongoing research initiative. Twins and their family members (siblings, offspring, spouses) participate on a voluntary basis. As of 2019 over 250,000 twins and family members have registered with the NTR, and over 26,000 participants have provided DNA samples for genome-wide SNP data. All adult participants have provided written informed consent, parents or primary caretakers have provided written informed consent for children. All data-collection protocols of the NTR are approved by the Medical Research Ethics Committee of the Amsterdam University Medical Centre and/ or the Ethical Review Board (VCWE) of the faculty of Behavior and Movement Science, Vrije Universiteit Amsterdam.

Genotyping, imputation and quality control

NTR participants were genotyped on the Affymetrix 6.0, Affymetrix Perlegen, Illumina Genomic Screening Array, Illumina Human660, or the Illumina Omni 1M array. Prior to imputation samples with a call rate below 90%, a mismatch between reported sex and biological sex, or an abnormal inbreeding F value (<-0.10 or >0.10) were removed. SNPs with a minor allele frequency (MAF) below 0.01, that deviate from Hardy-Weinberg Equilibrium ($p < 1*10^{-5}$), have a missing rate of > 5%, or have >20 Mendelian errors were removed. These quality control steps were performed on each of the genotyping platforms independently. After this initial round of quality control SNPs were imputed to the Genome of the Netherlands (GONL, The Genome of the Netherlands Consortium, 2014). The resulting genetic data was filtered based on the same conditions mentioned earlier, and an additional filter to drop SNPs with a low imputation quality ($R^2 < 0.90$). The resulting dataset was then imputed a second time using the Haplotype Reference Consortium (HRC) version 1.1. After a final round of quality control, genetic principal components were generated from this dataset, and ethnic outliers based on these components were removed.

Analysis

Siblings were identified using genetic relatedness to ensure only full biological siblings were included. Both an additive genetic, and dominance genetic relatedness matrix were computed for all participants. Pairs of participants with an additive genetic relatedness between 0.475 and 0.625, as

well as a dominance genetic relatedness between 0.125 and 0.375 were identified as biological siblings. For all phenotypes, except height and BMI, where multiple observations were available the last available observation was used. For height and BMI a direct measure was preferred over self-report, and the last available self-report was only used if no direct measure was available. If multiple direct measures of height or BMI were available, the last direct measure was used. Genetic analyses were performed using the scripts provided, with minor changes to fit local data storage formats. Age at survey completion, or measurement, of the included observation, sex, genotype platform and the first 20 principal components were used as covariates.

Acknowledgements

We would like to thank all members of twin families registered with the Netherlands Twin Register for their continued support of scientific research. NTR acknowledges past funding from the Netherlands Organization for Scientific Research (NWO) and The Netherlands Organisation for Health Research and Development (ZonMW) grants 904-61-090, 985-10-002, 912-10-020, 904-61-193,480-04-004, 463-06-001, 451-04-034, 400-05-717, Addiction-31160008, 016-115-035, 481-08-011, 400-07-080, 056-32-010, Middelgroot-911-09-032, OCW_NWO Gravity program –024.001.003, NWO-Groot 480-15-001/674, Center for Medical Systems Biology (CSMB, NWO Genomics), NBIC/BioAssist/RK(2008.024), Biobanking and Biomolecular Resources Research Infrastructure (BBMRI –NL, 184.021.007 and 184.033.111), X-Omics 184-034-019; Spinozapremie (NWO- 56-464-14192), KNAW Academy Professor Award (PAH/6635) and University Research Fellow grant (URF) to DIB; Amsterdam Public Health research institute (former EMGO+) , Neuroscience Amsterdam research institute (former NCA) ; the European Community's Fifth and Seventh Framework Program (FP5- LIFE QUALITY-CT-2002-2006, FP7- HEALTH-F4-2007-2013, grant 01254: GenomEUtwin, grant 01413: ENGAGE and grant 602768: ACTION); the European Research Council (ERC Starting 284167, ERC Consolidator 771057, ERC Advanced 230374), Rutgers University Cell and DNA Repository (NIMH U24 MH068457-06), the National Institutes of Health (NIH, R01D0042157-01A1, R01MH58799-03, MH081802, DA018673, R01 DK092127-04, Grand Opportunity grants 1RC2 MH089951, and 1RC2 MH089995); the Avera Institute for Human Genetics, Sioux Falls, South Dakota (USA). Part of the genotyping and analyses were funded by the Genetic Association Information Network (GAIN) of the Foundation for the National Institutes of Health. Computing was supported by NWO through grant 2018/EW/00408559, BiG Grid, the Dutch e-Science Grid and SURFSARA.

References

1. Ligthart, L., Van Beijsterveldt, C., Kevenaar, S., De Zeeuw, E., Van Bergen, E., Bruins, S., . . . Boomsma, D. (2019). The Netherlands Twin Register: Longitudinal Research Based on Twin and Twin-Family Designs. Twin Research and Human Genetics, 22(6), 623-636. doi:10.1017/thg.2019.93

2. The Genome of the Netherlands Consortium., Francioli, L., Menelaou, A. et al. Whole-genome sequence variation, population structure and demographic history of the Dutch population. Nat Genet 46, 818–825 (2014). https://doi.org/10.1038/ng.3021

Orkney Complex Disease Study (ORCADES)

Overview

The Orkney Complex Disease Study (ORCADES) is a family-based, cross-sectional study that seeks to identify genetic factors influencing cardiovascular and other disease risk in the isolated archipelago of the Orkney Isles in northern Scotland (McQuillan et al., 2008). Genetic diversity in this population is decreased compared to Mainland Scotland, consistent with the high levels of endogamy historically. 2078 participants aged 16-100 years were recruited between 2005 and 2011, most having three or four grandparents from Orkney, the remainder with two Orcadian grandparents. Fasting blood samples were collected and many health-related phenotypes and environmental exposures were measured in each individual. All participants gave written informed consent and the study was approved by Research Ethics Committees in Orkney and Aberdeen (North of Scotland REC).

Acknowledgements

The Orkney Complex Disease Study (ORCADES) was supported by the Chief Scientist Office of the Scottish Government (CZB/4/276, CZB/4/710), a Royal Society URF to J.F.W., the MRC Human Genetics Unit quinquennial programme “QTL in Health and Disease”, Arthritis Research UK and the European Union framework program 6 EUROSPAN project (contract no. LSHG-CT-2006-018947). DNA extractions were performed at the Edinburgh Clinical Research Facility, University of Edinburgh. We would like to acknowledge the invaluable contributions of the research nurses in Orkney, the administrative team in Edinburgh and the people of Orkney.

References

1. McQuillan R, *et al.* (2008) Runs of homozygosity in European populations, *Am J Hum Genet* 83: 359-372.

QIMR Berghofer Medical Research Institute (QIMR)

Overview

Phenotypic data were collected during a series of longitudinal studies of Australian twins and their families. The content and details of data collection have been previously described 1–4. These studies were approved by the QIMR Berghofer Medical Research Institute Human Research Ethics Committee and the storage of the data follows national regulations regarding personal data protection. All participants provided informed consent.

Genotyping, imputation and quality control

Samples were genotyped using multiple Illumina HapMap, Omni, and GSA arrays. Pre-imputation quality control included excluding SNPs based on genotype call rate <95%, HWE P<10-6, MAF = 0, GenTrain Score < 0.6 [if data available], Mean GenCall Score < 0.7 [older Illumina array families only], unique position and strand alignment in a BLAST search, and sex-chromosome specific filters (female genotyping rate >1% for chr Y (not relevant); male heterozygosity >1% for chr X), and individuals based on sample call rate <99% and gender mismatch. Pre-phasing and imputation to the 1000G Phase3 v5 reference panel was performed using Eagle v2.4 (phased output) on the Michigan server. After imputation, SNPs were excluded based on MAF <0.001 and a minimum MAC=5. Genetic ancestry outliers were also excluded.

Analysis

Analyses were performed with the scripts supplied with this project using age, sex and 20 principal components as covariates.

Acknowledgements

We greatly thank the twins and their families for their participation. Thanks also to Grant Montgomery and his team for DNA collection and processing and to Scott Gordon for quality control and imputation of the genomic data. Data collection in the Australian sample was made possible by multiple grants from National Health and Medical Research Council and the Australian Research Council.

References

1. Gillespie N, Kirk KM, Heath AC, Martin NG, Hickie I. Somatic distress as a distinct psychological dimension. Social Psychiatry and Psychiatric Epidemiology. 1999;34(9):451–458.
2. Kirk KM, Birley AJ, Statham DJ, Haddon B, Lake RI, Andrews JG, et al. Anxiety and depression in twin and sib pairs extremely discordant and concordant for neuroticism: prodromus to a linkage study. Twin research : the official journal of the International Society for Twin Studies. 2000;3(4):299.
3. Treloar SA, Martin NG, Bucholz KK, Madden PAF, Heath AC. Genetic influences on post-natal depressive symptoms: findings from an Australian twin sample. Psychological Medicine. 1999;29(3):645–654.
4. Wright MJ, Martin NG. The Brisbane Adolescent Twin Study: outline of study methods and research projects. Australian Journal of Psychology. 2004;56:65–78.

Swedish Twin Registry

Overview

The Swedish Twin Registry (STR) Aging samples included twins from several STR-based sub-studies: TwinGene (Zagai, Lichtenstein, Pedersen, & Magnusson, 2019), the Swedish Adoption Twin Study of Aging (SATSA) (Finkel & Pedersen, 2004), Aging in Women and Men (GENDER) (Gold, Malmberg, McClearn, Pedersen, & Berg, 2002), and Origins of Variance in the Oldest Old: Octogenarian Twins (OCTO-Twin) (McClearn et al., 1997), the Study of Dementia in Swedish Twins (HARMONY) (Gatz et al., 2005) and the Young Adult Twins in Sweden Study (YATSS) (Zagai, Lichtenstein, Pedersen & Magnusson, 2019).

Aging Sample I included 2764 dizygotic twins from the same pairs. TwinGene was collected between 2004 and 2008 with a total of 12 591 individuals. Phenotypic data, including coronary heart disease (CHD), Type 2 Diabetes (T2D) and stroke, were collected by self-reported questionnaire. In addition, diagnostic coding (ICD10) for coronary heart disease, diabetes and stroke were obtained from the Swedish National Patient Register up until 2010. Systolic blood pressures (SBP) were measured in mm Hg. Blood samples of 50 ml was drawn from each individual by venipuncture at their local health-care facility and sent overnight to Karolinska University Laboratory. Low- and high-density lipoprotein (LDL and HDL, respectively), triglycerides (TG) and C-reactive protein (CRP) were measured by routine methods on semiautomated biochemistry analyzer (Beckman Coulter, CA). Hemoglobin A1c (HbA1C) was measured by high-liquid performance chromatography separation technique.

Aging Sample II, subset 1 included 850 dizygotic twins, including those from same-sex or opposite-sex pairs, with available genotyping on the Illumina Infinium Psych Array (Illumina San Diego, CA, USA), including SATSA, GENDER, Octo-Twin, and Harmony. Phenotypic data on height and BMI prioritized first available in-person measurements over self-reported values in the home study. Assayed triglycerides using standard methods were taken from the first available in-person measurements in the home study, and per protocol values were standardized to mean zero and standard deviation one. Educational attainment, ever smoker, and depressive symptoms were collected via first available surveys in the home study. Education was coded into ISCED units and then to year equivalents per protocol. Depressive symptoms were measured from the 20-item Center for Epidemiologic Studies Depression Scale (CES-D), and per protocol standardized to mean zero and standard deviation one. Covariates included sex and age when all participants were still alive (i.e., age in 1992), and the first 10 genetic principal components as covariates.

In Aging Sample I, Aging Sample II subset 2 and the YATSS cohort, an additional set of phenotypes were analyzed separately. Education and reproductive data (age at first birth, number of children) were taken from public registers held by Statistics Sweden (the LISA database and the Multigeneration register, respectively). Tobacco use, subjective wellbeing and depression (CESD) were available in surveys from STR. The CESD surveys were available in the YATSS, STAGE and STAGE1 surveys, while tobacco use was available also in the 1973 STR survey. Subjective wellbeing was taken from the STR SALTY survey. Height and BMI were measured by a physician either at conscription or at a checkup conducted by STR as part of the TwinGene study. Cognitive capacity was measured using military conscription tests. All variables were prepared per protocol and analyzed with age in 2019, sex and the first 20 genetic principal components as covariates.

Informed consents were obtained from all participants. The different study collections were approved separately by the regional ethical review board in Stockholm and in Uppsala.

Genotyping, imputation and quality control

DNA was extracted from whole blood. For Aging Sample I, the serum was stored in liquid nitrogen. One 7ml EDTA tube of whole blood was stored in -80°C while a second 7ml EDTA tube of blood was used for DNA extraction. For both Aging Samples I and II, a 7ml EDTA tube of blood was used for DNA extraction using the Puregene extraction kit (Gentra systems, Minneapolis, USA). After extraction, the DNA was subsequently stored at -20°C.

Aging Sample I was genotyped on the Illumina OmniExpress bead chip array (700K). The quality controls for the set of analyses on the biological phenotypes included: individual missingness ≤0.03, genotype missingness ≤0.03, minor allele frequency ≥0.01, Hardy-Weinberg equilibrium P≥107, no sex mismatch, no excess heterozygosity (individuals with an F-statistic beyond 5 SDs from the sample mean), and no cryptic (unknown) relatedness. The genotyped data were imputed to the Haplotype Reference Consortium (HRC). For the remaining variables, preimputation filters were <.05 individual missingness, <.02 genotype missingness, MAF>.01 and HWE>1e-5, and imputation was done to HRC r1.1 using minimac4 on the Michigan imputation server (European reference panel).

Samples from the Aging Sample II, subset 1 were genotyped on the Illumina Infinium Psych Array (Illumina San Diego, CA, USA). Quality control steps included: excluding markers not mapped to a chromosome, MAF = 0, not meeting Hardy-Weinberg equilibrium (p < 1e-6), and sex or relatedness discrepancies. After removing ambiguous strand SNPs, samples were pre-phased using SHAPEIT v2.r837 and imputed to 1000 genomes phase 1 version 3 using IMPUTE2 version 2.3.2 with default parameters.

The Aging Sample II, subset 2 had preimputation filters set to <.05 individual missingness, <.02 genotype missingness, MAF>.01 and HWE>1e-5, and imputation was done to HRC r1.1 using minimac4 on the Michigan imputation server (European reference panel).

The YATSS sample was genotyped using the Illumina GSA array. Preimputation filters were set to <.05 individual missingness, <.02 genotype missingness, MAF>.0001 and HWE>1e-5, and imputation was done to HRC r1.1 using minimac4 on the Michigan imputation server (European reference panel).

Analysis

Phenotypic data preparation was performed in R-3.6.3 for biological phenotypes in Aging Sample I and SAS 9.4 (SAS, Inc., Cary, NC) for Aging Sample II, subset 1. Preparation of genotype data and analyses were performed using the provided study scripts.

Aging Sample I, Aging Sample II subset 2 and YATSS – Analyses were performed using the provided scripts including age in 2019, sex and 20 principal components as covariates.

Aging Sample II subset 1 – Analyses were performed using the provided scripts including age when all participants were still alive (i.e., age in 1992), sex and 10 principal components as covariates.

Acknowledgements

For STR Aging Sample II subset 1, PsychChip GWAS genotyping and collaborative work supported in part by the National Institutes of Health/National Institute on Aging grants R01 AG037985, R01 AG059329, and R01 AG060470 and DNA extraction by grants R01 AG17561 and R01 AG028555. Harmony was supported by grant R01 AG08724. OCTO-Twin was supported by grant R01 AG08861. Gender was supported by the MacArthur Foundation Research Network on Successful Aging, The Axel and Margaret Axson Johnson's Foundation, The Swedish Council for Social Research, and the Swedish Foundation for Health Care Sciences and Allergy Research. SATSA was supported by grants R01 AG04563, R01 AG10175, the John D. and Catherine T. MacArthur Foundation Research Network on Successful Aging, the Swedish Council For Working Life and Social Research (FAS) (97:0147:1B,

2009-0795) and Swedish Research Council (825-2007-7460, 825-2009-6141). The research was also supported by Riksbankens Jubileumsfond (P18-0782:1) and the Swedish Research Council (2019-00244).

We acknowledge The Swedish Twin Registry for access to data. The Swedish Twin Registry is managed by Karolinska Institutet and receives funding through the Swedish Research Council under the grant no 2017-00641. The content of this manuscript is solely the responsibility of the authors and does not necessarily represent the official views of the National Institutes of Health/National Institute on Aging.

References

1. Finkel, D., & Pedersen, N. (2004). Processing Speed and Longitudinal Trajectories of Change for Cognitive Abilities: The Swedish Adoption/Twin Study of Aging. *Neuropsychol Dev Cogn B Aging Neuropsychol Cogn, 11*(2), 325-345. doi:10.1080/13825580490511152

2. Gatz, M., Fratiglioni, L., Johansson, B., Berg, S., Mortimer, J. A., Reynolds, C. A., Fiske, A., & Pedersen, N. L. (2005). Complete ascertainment of dementia in the Swedish Twin Registry: the HARMONY study. *Neurobiology of Aging, 26*(4), 439-447. doi:10.1016/j.neurobiolaging.2004.04.004

3. Gold, C. H., Malmberg, B., McClearn, G. E., Pedersen, N. L., & Berg, S. (2002). Gender and health: a study of older unlike-sex twins. *J Gerontol B Psychol Sci Soc Sci, 57*(3), S168-176. Retrieved from http://www.ncbi.nlm.nih.gov/pubmed/11983743

4. McClearn, G. E., Johansson, B., Berg, S., Pedersen, N. L., Ahern, F., Petrill, S. A., & Plomin, R. (1997). Substantial genetic influence on cognitive abilities in twins 80 or more years old. *Science, 276*(5318), 1560-1563. Retrieved from http://www.ncbi.nlm.nih.gov/pubmed/9171059

5. Zagai, U., Lichtenstein, P., Pedersen, N. L., & Magnusson, P. K. E. (2019). The Swedish Twin Registry: Content and Management as a Research Infrastructure. *Twin Research and Human Genetics, 22*(6), 672-680. doi:PII S1832427419000999

The Twins Early Development Study (TEDS)

Overview

The Twins Early Development Study (TEDS) is a multivariate, longitudinal study of >16,000 twin pairs representative of England and Wales, recruited 1994–1996 (Rimfeld et al. 2019). The demographic characteristics of TEDS participants and their families closely match those of families in the UK. Written informed consent was obtained from parents prior to data collection and from TEDS participants themselves past the age of 18. Current analyses were conducted on a sub sample of dizygotic (DZ) twin pairs with genome-wide genotyping and phenotypic data.

Genotyping, imputation and quality control

Two different genotyping platforms were used because genotyping was undertaken in two separate waves. AffymetrixGeneChip 6.0 SNP arrays were used to genotype 3,665 individuals. Additionally, 8,122 individuals (including 3,607 DZ co-twin samples) were genotyped on Illumina HumanOmniExpressExome-8v1.2 arrays. After quality control, 635,269 SNPs remained for AffymetrixGeneChip 6.0 genotypes, and 559,772 SNPs for HumanOmniExpressExome genotypes.

Genotypes from the two platforms were separately phased and imputed into the Haplotype Reference Consortium (release 1.1) through the Sanger Imputation Service before merging. Genotypes from a total of 10,346 samples (including 3,320 DZ twin pairs and 7,026 unrelated individuals) passed quality control, including 3,057 individuals genotyped on Affymetrix and 7,289 individuals genotyped on Illumina. The identity-by-descent (IBD) between individuals was < 0.05 for 99.5% in the merged sample excluding the DZ co-twins (range = 0.00 – 0.12) and ranged between 0.36 and 0.62 for the DZ twin pairs (mean = 0.49). There were 7,363,646 genotyped or well-imputed SNPs (for full genotype processing and quality control details, see (Selzam et al. 2018)).

For the current analyses, we further restricted to variants with high confidence imputation accuracy (INFO score >.75) and minor allele frequency >1%. The final number of SNPs was 6,300,709.

Analysis

Current analyses were adjusted for sample plate and chip, in addition to 20 principal components. Principal components were derived from a subset of 39,353 common (MAF > 5%), perfectly imputed (INFO = 1) autosomal SNPs, after stringent pruning to remove markers in linkage disequilibrium (r2 > 0.1) and excluding high linkage disequilibrium genomic regions to ensure that only genome-wide effects were detected.

Acknowledgements

We gratefully acknowledge the ongoing contribution of the participants in the Twins Early Development Study (TEDS) and their families. TEDS is supported by a program grant to RP from the UK Medical Research Council (MR/V012878/1 and previously MR/M021475/1), with additional support from the US National Institutes of Health (AG046938).

References

1. Rimfeld K, Malanchini M, Spargo T, Spickernell G, Selzam S, McMillan A, et al. Twins Early Development Study: A Genetically Sensitive Investigation into Behavioral and Cognitive Development from Infancy to Emerging Adulthood. Twin Research and Human Genetics. 2019:1–6. https://doi.org/10.1017/thg.2018.75 PMID: 30661510

2. Selzam, S., McAdams, T.A., Coleman, J.R.I., et al. 2018. Evidence for gene-environment correlation in child feeding: Links between common genetic variation for BMI in children and parental feeding practices. PLoS Genetics 14(11), p. e1007757.

TwinsUK

Overview

TwinsUK is a cohort of volunteer adult twins from across the United Kingdom. The Registry was started in 1992 with the primary aim of assessment of heritability of osteoarthritis and osteoporosis in women. The success of early studies led to rapid evolution of the registry and it now incorporates about 12 000 twins, both male and female aged 18–103 years, studied for a whole range of clinical and behavioural traits.

Genotyping, imputation and quality control

5710 twins have undergone a genome-wide scan of either 317 000 single nucleotide polymorphism (SNP) markers (Illumina HumanHap300 Bead Chip) or 610, 000 SNPs (Illumina HumanHap610 Quad Chip). From these twins, 2840 participated in the first follow-up and 2545 in the second follow-up visits. The data was fully imputed using IMPUTE version 2 software, quality checked, and has been used in many international consortia for different phenotypes.

Acknowledgements

TwinsUK receives funding from the Wellcome Trust (212904/Z/18/Z) and European Union (H2020 contract #733100). TwinsUK and M.M. are supported by the National Institute for Health Research (NIHR)-funded BioResource, Clinical Research Facility and Biomedical Research Centre based at Guy’s and St Thomas’ NHS Foundation Trust in partnership with King’s College London. P.C. is founded by the European Union (H2020 contract #733100)

References

1. Moayyeri A, Hammond CJ, Valdes AM, Spector TD. Cohort Profile: TwinsUK and healthy ageing twin study. *Int J Epidemiol*. 2013;42(1):76-85. doi:10.1093/ije/dyr207

2. Moayyeri A, Hammond CJ, Hart DJ, Spector TD. The UK Adult Twin Registry (TwinsUK Resource). *Twin Res Hum Genet*. 2013;16(1):144-149. doi:10.1017/thg.2012.89

UK Biobank

Overview

UK Biobank is a large-scale prospective cohort study including 503,325 individuals aged between 38-73 years, who were recruited between 2006 and 2010 from across the United Kingdom. For the purposes of this study, we used a subsample of 40,210 siblings from 19,523 families. Full-siblings

were derived using UK Biobank provided estimates of pairwise identical by state (IBS) kinships (>0.5-21*IBS0, <0.7) and IBS0 (>0.001, <0.008), the proportion of unshared loci.

Genotyping, imputation and quality control

UK Biobank study participants (N= 488,377) were genotyped using the UK BiLEVE (N= 49,950) and the closely related UK Biobank Axiom™ Arrays (N= 438,427). Directly genotyped variants were pre-phased using SHAPEIT3 and imputed using Impute4 and the UK10K, Haplotype Reference Consortium and 1000 Genomes Phase 3 reference panels.

Analysis

Analyses were performed using the supplied scripts including birth year, sex and 20 principal components as covariates.

Acknowledgements

UK Biobank received ethical approval from the Research Ethics Committee (11/NW/0383). Access to UK Biobank data was granted as part of application 15825 (PI: Dr Philip Haycock).

References

Bycroft C. et al (2018) The UK Biobank resource with deep phenotyping and genomic data. *Nature*.

<u>Viking Health Study – Shetland (VIKING I)</u>

Overview

The Viking Health Study - Shetland (VIKING) is a family-based, cross-sectional study that seeks to identify genetic factors influencing cardiovascular and other disease risk in the population isolate of the Shetland Isles in northern Scotland (Kerr et al., 2019). Genetic diversity in this population is decreased compared to Mainland Scotland, consistent with the high levels of endogamy historically. 2105 participants were recruited between 2013 and 2015, most having at least three grandparents from Shetland. Fasting blood samples were collected and many health-related phenotypes and environmental exposures were measured in each individual. All participants gave informed consent and the study was approved by the South East Scotland Research Ethics Committee.

Acknowledgements

The Viking Health Study – Shetland (VIKING) was supported by the MRC Human Genetics Unit quinquennial programme grant "QTL in Health and Disease". DNA extractions and genotyping were performed at the Edinburgh Clinical Research Facility, University of Edinburgh. We would like to acknowledge the invaluable contributions of the research nurses in Shetland, the administrative team in Edinburgh and the people of Shetland.

References

Kerr S. *et al.* (2019) An actionable KCNH2 Long QT Syndrome variant detected by sequence and haplotype analysis in a population research cohort. *Sci Rep* 9: 10964.

Phenotype definitions

Phenotype definitions were suggested in the analysis plan, based on previous GWAS.

Height

The participants height while standing measured in centimetres. If height has been recorded with greater precision, round to the nearest centimetre. Where height data have been collected from multiple sources, preference is that measures from direct assessments of cohort participants are used above self-reported height.

The meta-analysis units for height are centimetres.

BMI

Please calculate BMI as weight in kilograms divided by standing height in metres squared (BMI = kg/m2). Where weight has been collected with greater precision than kilograms, please round to the nearest kilogram. Where height has been collected with greater precision than centimetres (0.01 metre), please round to the nearest centimetre. Where weight and height data have been collected from multiple sources, our preference is that measures from direct assessments of cohort participants are used above self-reported measures.

The meta-analysis units for BMI are kg/m^2.

Waist-hip ratio (WHR), adjusted for BMI

Please calculate waist-hip ratio adjusted for BMI by residualizing using linear regression. Waist and hip measures should be rounded to the nearest centimetre. Where multiple waist and hip measures are available our preference is for direct or clinic measurements above self-report. Please ensure that the waist-hip measurements and BMI were made concurrently. If there are multiple measurements over time, please choose the measurement occasion with the least missing data across your cohort.

The meta-analysis used untransformed WHR GWAS data.

Educational attainment

For years of education, completed academic qualifications should be mapped to levels of the International Standard Classification of Education (ISCED) and converted to the corresponding completed years of education required for completion of the qualification in US years of schooling. If ISCED levels 5 and 6 cannot be distinguished, please set values for those with tertiary education as 20 years of schooling.

The meta-analysis used standardised measures of years in full-time education (SD = 1).

Systolic blood pressure (SBP)

The participants systolic blood pressure adjusted for treatment with anti-hypertensives. Please use the time point that has the least missing data across your sample. Please replace missing values with values from other measurement occasion if multiple time points are available. If multiple measurements of blood pressure were taken at each clinic visit, please use the average of the measurements. If some participants have been treated with treated with anti-hypertensive medication, please add 10mmHg to the blood pressure of individuals who are on treatment at the time of measurement.

The meta-analysis units for SBP are mmHg.

Cognitive ability (performance on cognitive tests)

A general cognitive function score will be created, where appropriate data are available in the sample, by Principal Component Analysis (PCA) using at least 3 cognitive tests that assess different cognitive domains. Only one score should be used from each cognitive test. The tests should not

include the clinical cognitive assessments that are used as screening instruments for dementia (e.g., MMSE). The score to be created and used for the genetic analysis will be the first unrotated principal component. The general cognitive function score from the PCA (first unrotated principal component) should be saved as a standardized variable (mean = 0; SD = 1). The variable must be in the direction of higher positive scores indicating better general cognitive performance.

The meta-analysis used standardised measures of cognitive ability (SD = 1).

Smoking behaviour

For each smoking phenotype only include information on cigarette smoking behaviour. Do not include information about pipes, cigars, or other forms of tobacco use.

For ever smoker, a binary measure recording those who report ever being a regular smoker in their life, either current or former.

For smoking intensity, the average number of cigarettes smoked per day as a current or former smoker (set values for non-smokers to missing, not zero). If numbers of cigarettes per day have been recorded as categorical (e.g. 1-5 cigarettes per day), set value as the mid-point of the range (e.g. 2.5). Please remove extreme outliers (e.g. 5 S.D. away from the mean).

The meta-analysis used risk increase (ever smoking) and untransformed cigarettes per day (smoking intensity) data.

Alcohol consumption

The average number of alcoholic units consumed per week as reported by the participant, aggregated across all types of alcohol. If numbers of drinks have been recorded as categorical (e.g. 1-5 drinks per week), set value as the mid-point of the range (e.g. 2.5). Please remove extreme outliers (e.g. 5 S.D. greater than the mean).

The meta-analysis used untransformed units of alcohol consumption.

Depressive symptoms

Depression is a highly heterogeneous disorder with many clinical presentations. The diagnosis requires a distinct change of mood, characterized by sadness or irritability, accompanied by psychophysiological changes, such as disturbances in sleep, appetite, or sexual desire; constipation; loss of the ability to experience pleasure in work or with friends; crying; suicidal thoughts; and slowing of speech and action.

A great variety of rating scales have been developed to assess symptoms of depression. The scales differ in the number of items included and with respect to the types of symptoms assessed: mood symptoms (depressed mood and irritability), behavioural symptoms (suicide and anhedonia), somatic symptoms (appetite disturbance, sleep disturbance, low energy, and psychomotor retardation or agitation), cognitive symptoms (hopelessness and worthlessness), and concentration symptoms (poor concentration and decision-making). Frequently used scales include: the Hamilton Rating Scale for Depression (HRSD), the Beck Depression Inventory (BDI), the Center for Epidemiologic Studies Depression Scales (CES-D), the Quick Inventory of Depressive Symptoms (QIDS), and the Self-Rating Depression Scale (SDS). In MoBa (one of the largest cohorts) depressive symptoms were measured by the Short Mood and Feelings Questionnaire (SMFQ) in adolescents, and by the Hopkins Symptom Checklist (H-SCL) in adults.

If multiple rating scales of depressive symptoms are available, select the scale with most items (tapping most symptom domains). Please sum the items and treat the final summed variable as a continuous measure of depression symptoms. Please ensure that measures are coded so that higher values represent higher symptom levels. Please standardize the score to have mean zero standard deviation one.

The meta-analysis used standardised measures of depressive symptoms (SD = 1).

Subjective wellbeing

Reporting of subjective wellbeing is likely to be highly variable across studies. Our preference is for subjective wellbeing to have been measured using a battery of questions. If your study has done this, please sum responses and treat this final summed variable as a continuous measure of subjective wellbeing. If data on subjective wellbeing are only available from a single question reported on a Likert scale, please treat the response variable as continuous. Please ensure that measures are coded so that higher values represent higher levels of subjective wellbeing.

The meta-analysis used standardised measures of subjective wellbeing (SD = 1).

Neuroticism

Neuroticism is a fundamental domain of personality functioning and structure. The personality trait refers to a lack of emotional stability; stress vulnerability; the tendency to experience intense negative emotions, affects, and cognitions; and impulsive behaviours under emotional strain. Neuroticism can be validly measured from age 6-7 years. The Hierarchical Personality Inventory for Children is one of few instruments specifically designed to assess neuroticism in children. For adults, scales include the NEO-Personality Inventory, the Eysenck Personality Questionnaire, and various scales using Big-Five factor markers from the International Personality Item Pool (IPIP- including the 300-item representation of the Revised NEO Personality Inventory). Most modern neuroticism scales show strong overlap in item content (although they consist of different items), and the sum scores correlate highly across inventories. Scales usually agree on lower-order traits (e.g. anxiety-withdrawal, depression-unhappiness, vulnerability-stress reaction). However, there is less agreement whether aggression, impulsivity, inferiority, and dependency belong to the neuroticism domain.

Please sum the neuroticism items and treat the final summed variable as a continuous measure. The scale should be standardized with mean=0 and SD=1. Please ensure that higher values on the scale represent higher levels of neuroticism.

The meta-analysis used standardised measures of neuroticism (SD = 1).

Number of biological children

Restricted to study participants who have reached the end of their reproductive window, defined as over the age of 45 if female and over the age of 55 if male.

Number (of children) ever born (NEB)

NEB can be treated as a continuous measure that has been asked directly or can be imputed from several survey questions (such as pregnancy histories). A standard question within most surveys asks:

How many children have you given birth to?

Another variant is:

How many children do you have?

In most cases it is also possible to distinguish between biological (live born or stillborn), adopted or stepchildren. When this information is available, we will refer to **live born biological children**. In some surveys, the birth date of each child is asked, enabling a calculation of NEB. In many surveys, this question has been adapted for male and female subjects and in several cases only women have been asked. Sometimes, there is a filter in the survey, asking whether the respondent has any

children. For twin studies please include a count of 2 for all live born biological children. Individuals are eligible if they meet the following criteria:

1. They were assessed for NEB at least at age 45 for women, age 55 for men;
2. Those who have both given birth to a child (parous) and those who have not (nulliparous);
3. All relevant covariates (year of birth) are available for the individual;
4. They were successfully genotyped genome-wide (recommended individual genotyping rate > 95%);
5. They passed the cohort-specific standard quality controls, e.g. excluding individuals who are genetic outliers in the cohort.

The meta-analysis used the untransformed number of biological children.

Age at first birth (AFB)

AFB can be treated as a continuous measure, which has generally been asked directly or can be imputed from several survey questions (such as date of birth respondent and date of birth of first child). The most common question is:

How old were you when you had your first child?

Another variant is:

What is the date of birth of your first child?

In the case of the latter, you can simply create a new AFB variable by subtracting the date of birth of the first child from the date of birth of the respondent. In many surveys, this question has been adapted for male and female subjects and in some cases only women have been asked. Individuals are eligible if they meet the following criteria:

a. They were assessed for AFB and have given birth to a child (parous); both for females and for males.
b. All relevant covariates (year of birth) are available for the individual;
c. They were successfully genotyped genome-wide (recommended individual genotyping rate > 95%);
d. They passed the cohort-specific standard quality controls, e.g., excluding individuals who are genetic outliers in the cohort.

The meta-analysis used untransformed age at first birth.

C-reactive protein (CRP)

Please measured serum CRP in mg/L by using standard laboratory techniques and transform the values by natural log. Please exclude individuals with auto-immune diseases, individuals taking immune-modulating agents (if this information was available), and individuals with CRP values 4 SD or more away from the mean from CRP analyses (i.e. set to missing). Please normalize to mean zero and SD one.

The meta-analysis used standardised measures of CRP (SD = 1).

HbA1c

Trait are “raw” untransformed HbA1c values in % of hemoglobin, without rank normalization. Set all individuals with Diabetes (T2D, T1D) to missing ('diagnosed', on diabetes treatment (oral and insulin)

or fasting plasma glucose (FPG) >=7 mmol/L). If FPG is NOT available, set individuals with 2hr-hour glucose >= 11.1 mmol/L and/or HbA1c >= 6.5%. Set participants with major blood abnormalities (thalassemia, sickle cell anemia, etc) or those who have had a blood transfusion in the previous 2-3 months. Definition taken from Wheeler et al. (2017).

The meta-analysis used standardised measures of HbA1c (SD = 1).

Lipids

The participants untreated LDL-cholesterol, HDL-cholesterol and triglyceride levels. Please replace missing values with values from other measurement occasion if multiple time points are available. If multiple measurements of cholesterol were taken at each clinic visit, please use the average of the measurements. Please normalise to mean zero standard deviation one.

The meta-analysis used standardised measures of LDL, HDL and TG (SD = 1).

Lung function (FEV1/FEV1FVC)

Restrict dataset to those individuals with no missing data for the ever smoking or never smoking variable and to those with complete data on both forced expiratory volume (FEV1) and forced vital capacity (FVC). Undertake linear regression of age, age squared, sex, height on FEV1 and use residuals for all subsequent analyses. Transformation would be taken once for each trait (FEV1 and FEV1/FVC ratio) and used for all analyses (including subgroups). Transform residuals to ranks and then to normally distributed z-scores. These inverse-normal transformed residuals are then used as the phenotype for association testing under an additive genetic model.

Never-smokers only: Repeat analysis as for 1

Ever-smokers only: Repeat analysis as for 1

Repeat the above for outcomes: **FEV1/FVC ratio**

The meta-analysis used standardised measures of FEV1 and FEV1/FVC ratio (SD = 1).

Age at menarche

Age at menarche should be treated as a continuous measure in years, which has generally been asked directly of study participants. The most common question is:

How old were you when you had your first menstrual period?

In the event of responses being recorded in multi-year categories please set the response as the year midpoint (For example, the response category 8-9 would be recoded as 8.5). See Day et al (2017) for further information.

Day et al. Genomic analyses identify hundreds of variants associated with age at menarche and support a role for puberty timing in cancer risk. 2017. Nature Genetics, 49:834–841.

The meta-analysis used untransformed age at menopause.

Age at menopause

Age at menopause should be treated as a continuous measure in years, which has generally been asked directly of study participants. The most common question is:

At what age did your natural periods cease?

If the information is available, women who had radiation or chemotherapy or surgically induced menopause should be excluded from the analysis. In line with He et al (2007) we suggest that women who report ages at menopause earlier than age 40 or later than age 60 should be excluded.

He et al. Genome-wide association studies identify loci associated with age at menarche and age at natural menopause. 2009. Nature Genetics, 41:724–728.

The meta-analysis used untransformed age at menarche.

Estimated glomerular filtration rate (eGFR)

1) If necessary correct the serum creatine measurements. This will depend on the measurement method (example given for HUNT)

- In HUNT serum creatine was measured using the Isotope Dilution Mass Spectroscopy Method (IDMS).

a) For HUNT2 either use corrected serum creatine measurement or apply the correction formula (1.11 x s-creatinine HUNT2 -27.4).

b) For HUNT3, correct the serum creatine measurement using the formula (0.889 x s-creatinine HUNT3 +12.6) x 1.11 -27.4)

2) Use corrected serum creatine to calculate eGFR by CKD-EPI (4-equations) model. Here are the 4 equations:

a) For "Female" & SeCrea <=61.9: eGFR= (144 * (SeCrea/61.9)^-0.329 * (0.993)^age)

b) For "Female" & SeCrea2>61.9: eGFR= (144 * (SeCrea/61.9)^-1.209 * (0.993)^age)

c) For "Male" & SeCrea<=79.6: eGFR= (141 * (SeCrea/79.6)^-0.411 * (0.993)^age)

d) For "Male" & SeCrea>79.6: eGFR= (141 * (SeCrea/79.6)^-1.209 * (0.993)^age)

The meta-analysis used untransformed eGFR.

Physical activity

Moderate-to-vigorous intensity leisure time activity (more vs. less than 20 min/week)

Equal to or more than > 20 minutes of “strenuous sports” and “other exercise” combined (dichotomized)

Equal to or more than > 20 minutes of physical activity => METs 3 (dichotomized)

The meta-analysis used risk increase in the binary physical activity measure.

Supplementary Methods

Meta-analysis quality control

Further quality control was performed prior to meta-analyses. We used phenotype-specific genotype counts (e.g., from the sample with height data in a study) to exclude variants missing in more than 10% of samples. We found some evidence that low frequency variants in small sample sizes may have inflated test statistics in regression models. We randomly selected two sets of 250 sibship (~500 individuals) in UK Biobank and performed population and within-sibship GWAS. We found high levels of test statistic inflation with hundreds (population model) or thousands (within-sibship model) of genome-wide significant hits despite the small sample size. These variants were found to be overwhelmingly low frequency; the 99th percentile MAF for the genome-wide significant variants were 3.0%/2.3% in within-sibship model and 2.9%/4.6% in the population model. Therefore, we used phenotype-specific MAFs and study-level imputation quality (INFO scores) to perform additional stringent quality control on the GWAS data using the following cut-offs for INFO and MAF; fewer than 1,000 individuals (MAF < 0.1, INFO < 0.8); 1,000-3,000 individuals (MAF < 0.05, INFO < 0.5); 3,000-5,000 individuals (MAF < 0.03, INFO < 0.5); 5,000-10,000 individuals (MAF < 0.02, INFO < 0.3); and more than 10,000 individuals (MAF < 0.01, INFO < 0.3).

Applying LD score regression to within-sibship data

LDSC is a widely used method that can be applied to GWAS summary data to estimate heritability and genetic correlation [1, 2]. Central to the method is that LDSC can detect and control for confounding (which is not correlated with LD scores) in GWAS data such as from cryptic relatedness and population stratification. The LDSC ratio, a function of the LDSC intercept unrelated to statistical power, is a measure of the proportion of association signal that is due to confounding. Notably the LDSC ratio will not identify sources of association that are correlated with LD scores such as indirect genetic effects or assortative mating as confounding. We therefore loosely interpret the LDSC ratio as a measure of confounding as it will not identify all sources of confounding. In this work, we apply LDSC to estimate SNP heritability and genetic correlation using the population and within-sibship GWAS data, so we investigated the LDSC intercept/ratio estimates from these data.

In theory, within-sibship data should be less susceptible to confounding than population data as it more effectively controls for population stratification than including principal components. To investigate this in practice, we used LDSC to estimate confounding in meta-analysis summary data for 25 phenotypes. Summary data were harmonised using the LDSC munge_sumstats.py function. LDSC intercepts and ratios were estimated using the harmonised data and the LDSC ldsc.py function with the precomputed European LD scores from the 1000 Genomes (Phase 3) reference panel. The LDSC ratio was used for comparisons between phenotypes and studies as it is not a function of statistical power. The LDSC ratio is calculated from the intercept (i) and the mean chi squared χ^2 as follows:

$$Ratio = \frac{i - 1}{mean(\chi^2) - 1}$$

LDSC confounding estimates varied across the 25 phenotypes in the within-sibship model. Confounding estimates were modest for height (10%, 95% C.I. [6%, 14%]) and BMI (9%, [2%, 16%]) while the estimate for educational attainment was imprecise (35%, [12%, 57%]). Across all

phenotypes in the within-sibship data, the median confounding estimate was 21% (Q1-Q3: 10%, 28%) but stronger conclusions are limited by imprecise estimates (**Supplementary Table 11**/ **Extended Data Figure 8**). The LDSC confounding estimates were higher using the population GWAS data (median 42%: Q1-Q3 35%, 56%) than both the within-sibship model and previous studies (**Supplementary Table 12)**. For example, the population model LDSC ratio estimates were higher for height (23%, [21%, 26%]), BMI (22%, [19%, 25%]) and educational attainment (41%, [37%, 45%]).

The observed non-zero confounding in the within-sibship model was unexpected because of the intuition that the within-sibship GWAS models are unlikely to be confounded. The LDSC ratios in the population GWAS were also higher than previous studies. We followed up these findings by evaluating the effects of LD score mismatch and cryptic relatedness on the LDSC ratios.

Evaluation of LD score mismatch

A large proportion of samples in the meta-analysis were from UK based studies such as UK Biobank and Generation Scotland, for which the LD scores, generated using 1000 Genomes project (phase 3) European samples (CEU, TSC, FIN, GBR), have been shown to fit reasonably well [1]. However, a large number of samples were from Scandinavian populations (HUNT, FinnTwin), where LD mismatch leading to elevated LDSC intercept/ratios has been previously discussed [1]. We investigated this possibility using empirical and simulated data.

We investigated variation in LDSC ratios across populations by comparing ratios for height across well-powered individual studies (N > 5,000): UK Biobank, HUNT, China Kadoorie Biobank (using default East Asian LD scores), Generation Scotland, DiscoverEHR, QIMR and FinnTwin. We found some evidence of heterogeneity between studies; ratio estimates were higher in Scandinavian studies compared to UK-based studies (**Extended Data Figure 9**). We also calculated within-sibship ratio estimates for BMI, SBP and educational attainment using UK Biobank summary data. UK Biobank estimates were largely consistent with zero confounding although confidence intervals were wide (**Supplementary Table 13**).

We performed simulations to evaluate potential mismatch between the Norwegian HUNT study and the default LD scores, which were generated using 1000 Genomes data. We used simulated phenotypes and real genotype data from UK Biobank and HUNT. We estimated the LDSC ratios as above, hypothesising that estimates higher than 0 are likely to reflect LD score mismatch because the phenotypes were simulated to not be influenced by confounders or common environmental terms (which could lead to cryptic relatedness).

Our process was as follows:

a) Select 1,000 HapMap3 SNPs at random.

b) Simulate beta weights for each SNP under a normal distribution with variance defined as a function of allele frequencies. The beta weight for SNP j was simulated as follows:

$Beta_{\,j} \sim N(0, 2p_j\,(1 - p_j))$ where p_j is the minor allele frequency of SNP j.

c) Generate polygenic scores for each individual using these weights.

d) Simulate phenotype with 30% of variation explained by polygenic score, with the rest of the variation random.

e) Run GWAS on the simulated phenotype.

In UK Biobank we used the Sibling GWAS pipeline on the same sample of siblings. In HUNT we used FastGWA [3] with a sparse GRM on a sample of 30,694 individuals not included in the sibling GWAS sample. The GWAS method and study sample is not particularly important in this context as there were no common environmental effects or confounders in the simulations.

f) Apply LDSC using EUR LD scores to estimate LDSC ratios.

From 10 simulations, the median LDSC ratio estimate was 0.05 (95% C.I. [-0.02, 0.12]) in the population model and 0.05 (95% C.I. [-0.07, 0.16]) in the within-sibship model in UK Biobank, consistent with minimal confounding. In contrast, the median ratio estimate in HUNT was 0.16 (95% C.I. [0.09, 0.23]) when using the default 1000 Genomes LD scores, highly suggestive of non-zero confounding. Using a HUNT-specific LD score reference panel generated using whole genome sequencing data, the median ratio estimate decreased to 0.11 (95% C.I. [0.04, 0.20]) but still suggested non-zero confounding. However, as detailed below, this did not affect the SNP h^2 estimates.

The combined findings from the empirical and simulated analyses suggest that LD score mismatch with the 1000 Genomes LD scores in HUNT and other studies likely contributed to inflated LDSC ratios in both population and within-sibship GWAS models.

Cryptic relatedness

One source of inflation in GWAS associations is cryptic relatedness; non-independence between close relatives in the study sample results which leads to inflated precision. In sibling GWAS models we clustered standard errors over sibships, but this clustering does not account for non-independence between related sibships, e.g., uncle/mother and two offspring. Inflated signal relating to cryptic relatedness may result in confounded signal, which is detected by the LD score intercept/ratio. In conventional population GWAS, close relatives are either removed or a mixed model is used to account for close relatives.

The HUNT Study population includes many second- and third-degree relatives. To investigate the extent to which cryptic relatedness may have impacted LDSC ratio estimates from the population model, we investigated the effect on the LDSC ratio of using a method that accounts for relatedness. We ran a conventional population GWAS of height using FastGWA [3], which accounts for close relatives using a sparse GRM (IBD > 0.05). We included age, sex, batch and the first 20 principal components as covariates. Using the GWAS summary data we then estimated the LDSC ratio using the 1000 Genomes reference panel and compared with previously described ratio estimates. We found that the FastGWA LDSC ratio (0.33; 95% C.I. [0.28, 0.39]) was substantially lower than the population model LDSC ratio (0.69; 95% C.I. [0.65, 0.73]) suggesting that cryptic relatedness was a source of inflation in the LDSC ratio for the population model.

Cryptic relatedness is an issue for non-family models but may not be an issue for within-family models. We performed simulations to investigate how cryptic relatedness would affect the standard errors of the population and within-sibship GWAS models.

Simulations included 3 generations (generations 1, 2 and 3), and we considered only a single genetic variant *G*. We assumed random mating across all generations and complete Mendelian inheritance for G. Individuals in generation 1 were all unrelated and after pairing randomly, each pair had 2 offspring (generation 2). Similarly, individuals in generation 2 paired randomly and had 2 offspring (generation 3). Generation 2 contained sibling pairs and Generation 3 contained first cousin quads (i.e., two pairs of siblings who are first cousins).

We simulated a common environmental term *C* for Generation 2, which was identical for the full siblings. In Generation 3, *C* was defined as the mean of parental *C* in Generation 2. We then simulated a normally distributed phenotype *P* in Generation 3 in which 30% of the variation was explained by *C* and the other 70% of variation was random. Note that *P* is not directly associated with *G*. We then performed regressions of the genetic variant on the phenotype using the population and within-sibship models, extracting the regression P-values. We repeated these simulations and regressions 10,000 times. We found that the type 1 error rate was inflated in the population model (5.84 %) (i.e., the false positive rate was higher than 5%) but not in the within-sibship model (4.94 %).

These findings suggest that the standard errors in the within-sibship model are not underestimated because of cryptic relatedness relating to common environmental effects shared between relatives. This, cryptic relatedness likely inflated LDSC ratios in the population models but not in the within-sibling data. Code for simulations on cryptic relatedness is available on GitHub (github.com/LaurenceHowe/SiblingGWASPost/blob/master/LDSCsimulations/CrypticRelatednessSims.R).

Within-sibship SNP heritability estimates - simulations

We used simulated data to validate the use of effective sample sizes and to explore the effects of LD score mismatch, and bias in the LDSC intercept on SNP heritability estimates. In the previously described simulations (in "evaluation of LD score mismatch") we also estimated SNP heritability alongside the LDSC ratios. In UK Biobank, the median SNP heritability across 10 simulations was 0.29 (95% C.I. [0.23, 0.34]) in the population model and 0.32 (95% C.I. [0.21, 0.42]) in the within-sibship model, highly comparable to the true simulated heritability of 0.30. In HUNT, SNP heritabilities were unbiased using both reference panels, but the median SNP heritability estimate was more precise using the HUNT LD scores (0.31; 95% C.I. [0.25, 0.38]) than the 1000 Genomes LD scores (0.31; 95% C.I. [0.21, 0.42]).

The simulated data suggests that LDSC can provide unbiased estimates of SNP heritability even in the presence of LD score mismatch. However, extensive simulations beyond the scope of this project are required to investigate this further.

Polygenic adaptation – sensitivity analyses

First, we evaluated the mean tSDS in the within-sibship model for a subset of independent variants strongly associated with height. We determined these variants by LD clumping the within-sibship meta-analysis GWAS data in PLINK v 2.0 ($r^2 < 0.001$, physical distance threshold = 10,000 kb, $P < 1\times10^{-5}$). Second, we used LDSC to estimate the genetic correlation between the SDS scores and the height GWAS data from the population and within-sibship models. The SDS input data was normalised (as above) SDS and we used the precomputed European LD scores from 1000 Genomes. Third, we also calculated spearman rank correlations between height and the SDS (as above) using summary data from individual studies (as opposed to the meta-analysis GWAS). We used studies with N > 4000, which were UK Biobank, HUNT, Generation Scotland, QIMR, Netherlands Twin Register, FinnTwin, Discover EHR and China Kadoorie Biobank and investigated both

population/within-sibship models. We then used a fixed effects model to meta-analyse the correlation estimates across the studies for the population and within-sibship models. Notably, the correlation estimate using only the UK Biobank WF summary data was inconclusive (r = 0.002; 95% C.I. -0.005, 0.010), consistent with a previous study [4], and correlation point estimates from individual studies were generally smaller than the meta-analysis GWAS estimates. This heterogeneity could relate to the increased number of samples in the meta-analysis, with a higher signal to noise ratio in the individual studies.

References

1. Bulik-Sullivan BK, Loh P-R, Finucane HK, Ripke S, Yang J, Patterson N, et al. LD Score regression distinguishes confounding from polygenicity in genome-wide association studies. Nature Genetics. 2015;47(3):291-5.
2. Bulik-Sullivan B, Finucane HK, Anttila V, Gusev A, Day FR, Loh P-R, et al. An atlas of genetic correlations across human diseases and traits. Nature Genetics. 2015;47(11):1236-41.
3. Jiang L, Zheng Z, Qi T, Kemper KE, Wray NR, Visscher PM, et al. A resource-efficient tool for mixed model association analysis of large-scale data. Nature Genetics. 2019.
4. Berg JJ, Harpak A, Sinnott-Armstrong N, Joergensen AM, Mostafavi H, Field Y, et al. Reduced signal for polygenic adaptation of height in UK Biobank. eLife. 2019;8. Epub 2019/03/22. doi: 10.7554/eLife.39725. PubMed PMID: 30895923.
