# Supplementary material for: Within-sibship genome-wide association analyses decrease bias in estimates of direct genetic effects
Source: Nat Genet. 2022 May 9;54(5):581–92. doi: 10.1038/s41588-022-01062-7 (PMC9110300; doi:10.1038/s41588-022-01062-7)
Supplement: Supplementary file 2 — Reporting Summary [file 41588_2022_1062_MOESM2_ESM.pdf]

nature research

Corresponding author(s): David R. Raleigh, MD, PhD

Last updated by author(s): Mar 11, 2022

Reporting Summary

Nature Research wishes to improve the reproducibility of the work that we publish. This form provides structure for consistency and transparency in reporting. For further information on Nature Research policies, see our Editorial Policies and the Editorial Policy Checklist.

Statistics

For all statistical analyses, confirm that the following items are present in the figure legend, table legend, main text, or Methods section.

n/a	Confirmed	
☐	☒	The exact sample size (*n*) for each experimental group/condition, given as a discrete number and unit of measurement
☐	☒	A statement on whether measurements were taken from distinct samples or whether the same sample was measured repeatedly
☐	☒	The statistical test(s) used AND whether they are one- or two-sided<br>*Only common tests should be described solely by name; describe more complex techniques in the Methods section.*
☐	☒	A description of all covariates tested
☐	☒	A description of any assumptions or corrections, such as tests of normality and adjustment for multiple comparisons
☐	☒	A full description of the statistical parameters including central tendency (e.g. means) or other basic estimates (e.g. regression coefficient) AND variation (e.g. standard deviation) or associated estimates of uncertainty (e.g. confidence intervals)
☐	☒	For null hypothesis testing, the test statistic (e.g. *F*, *t*, *r*) with confidence intervals, effect sizes, degrees of freedom and *P* value noted<br>*Give P values as exact values whenever suitable.*
☒	☐	For Bayesian analysis, information on the choice of priors and Markov chain Monte Carlo settings
☐	☒	For hierarchical and complex designs, identification of the appropriate level for tests and full reporting of outcomes
☐	☒	Estimates of effect sizes (e.g. Cohen's *d*, Pearson's *r*), indicating how they were calculated

Our web collection on statistics for biologists contains articles on many of the points above.

Software and code

Policy information about availability of computer code

Data collection

No software was used for data collection.

Data analysis

The open-source software, tools, and packages used for data analysis in this study, as well as the version of each program, were ImageJ (v2.1.0), R (v3.5.3 and v3.6.1), FASTQC (v0.11.9), HISAT2 (v2.1.0), featureCounts (v2.0.1), Seurat R package (v3.0.1), Harmony R package (v0.1), caret R package (v6.0-90), Rtsne R package (v0.15), PAMES R package (v2.6.2), CONICSmat R package (v1.0), DeepTools (v3.1.2), survival R package (v3.2-13), survAUC R package (v1.0-5), rms R package (v6.2-0), rpart R package (v4.1.16), DynNom R package (v5.0.1), DESeq2 (Bioconductor v3.10), SeSAMe (Bioconductor v3.10), minfi (Bioconductor v3.10), karyoplotR (Bioconductor v3.10), ConsensusClusterPlus (Bioconductor v3.10), and DiffBind (Bioconductor v3.10). No software was used for data collection. A methylation profile multi-class support vector machine (SVM) classifier was generated using the caret R package, and was deposited in the github repository abrarc/meningioma-svm (DOI:10.5281/enodo.6353877). In brief, a linear kernel SVM was constructed using training data comprising 75% of randomly selected samples from the discovery cohort (n=150) with 10-fold cross validation. 2,000 probes from each pre-processing pipeline were used as variables. The remaining 25% of samples from the discover cohort (n=50) were used to test the model, which performed with 97.9% accuracy when classifying samples into 3 SeSAMe groups (95% CI 89.2-99.9%, p<2.2x10-16). SVM classifiers for 3, 4, 5, or 6 minfi groups were generated using the same approach and performed with 91.8% (95% CI 80.4%-97.7%, p=4.69x10-9), 91.8% (95% CI 80.4%-97.7%, p=9.58x10-16), 93.8% (95% CI 82.8%-98.7%, p=2.98x10-16), and 93.6% (95% CI 82.5%-98.7%, p<2.2x10-16) accuracy, respectively. SVM classification and K-means consensus clustering of the validation cohort was performed with the same parameters as for the discovery cohort using the same probes in the validation cohort that were identified from the discovery cohort.

For manuscripts utilizing custom algorithms or software that are central to the research but not yet described in published literature, software must be made available to editors and reviewers. We strongly encourage code deposition in a community repository (e.g. GitHub). See the Nature Research guidelines for submitting code & software for further information.

Data

Policy information about availability of data

All manuscripts must include a data availability statement. This statement should provide the following information, where applicable:

- Accession codes, unique identifiers, or web links for publicly available datasets
- A list of figures that have associated raw data
- A description of any restrictions on data availability

DNA methylation (n=565), RNA sequencing (n=185), and single-cell RNA sequencing data (n=8 meningioma samples, n=2 dura samples) of new samples reported in this manuscript have been deposited in the NCBI Gene Expression Omnibus under the accession GSE183656 (https://www.ncbi.nlm.nih.gov/geo/query/acc.cgi?acc=GSE183656). Additional RNA sequencing data from previously reported meningiomas (n=15) from the discovery cohort are available under the accession GSE101638 (https://www.ncbi.nlm.nih.gov/geo/query/acc.cgi?acc=GSE101638). Whole exome sequencing, ChIP sequencing, and additional DNA methylation profiling data incorporated into this study were derived from previously reported and deposited meningiomas in GSE101638 (https://www.ncbi.nlm.nih.gov/geo/query/acc.cgi?acc=GSE101638), GSE139652 (https://www.ncbi.xyz/geo/query/acc.cgi?acc=GSE139652), and . The publicly available GRCh38 (hg38, https://www.ncbi.nlm.nih.gov/assembly/GCF_000001405.39/) and CRCh37.p13 datasets (hg19, https://www.ncbi.nlm.nih.gov/assembly/GCF_000001405.25/) were used in this study.

Field-specific reporting

Please select the one below that is the best fit for your research. If you are not sure, read the appropriate sections before making your selection.

☒ Life sciences ☐ Behavioural & social sciences ☐ Ecological, evolutionary & environmental sciences

For a reference copy of the document with all sections, see nature.com/documents/nr-reporting-summary-flat.pdf

Life sciences study design

All studies must disclose on these points even when the disclosure is negative.

Sample size	No statistical methods were used to predetermine clinical sample sizes, but our discovery and validation cohort sizes are similar or larger to those reported in previous publications. All experiments were performed with independent biological replicates (2-3 biological replicates for molecular or cell biology experiments, and 7+ biological replicates for animal experiments). In our experience and in the experience from previous publications, these samples sizes provide sufficient resolution to resolve biologically-meaningful differences between conditions tested using molecular, cellular, or animal techniques. To validate this approach, all experiments were repeated, and statistics were derived from biological replicates (rather than technical replicates). Biological replicates are indicated in each panel or figure legend. Data distribution was assumed to be normal, but this was not formally tested.
Data exclusions	No clinical, molecular, cellular, or animal data points were excluded from the analyses.
Replication	All experiments were performed with at least 3 biologic replicates. All attempts at replication were successful.
Randomization	This was a retrospective non-randomized study of human tumor samples with no intervention. All samples were interrogated equally. Thus, controlling for covariants among clinical samples is not relevant. Cells, organoids, and animals were randomized across experimental conditions, but pre-treatment tumor sizes and other potentially-confounding covariates were controlled across conditions before experimentation.
Blinding	Investigators were blinded to conditions during clinical data collection and analysis of mechanistic or functional studies. Bioinformatic analyses were performed blind to clinical features, outcomes, or molecular characteristics.

Reporting for specific materials, systems and methods

We require information from authors about some types of materials, experimental systems and methods used in many studies. Here, indicate whether each material, system or method listed is relevant to your study. If you are not sure if a list item applies to your research, read the appropriate section before selecting a response.

Materials & experimental systems

n/a	Involved in the study	
☐	☒	Antibodies
☐	☒	Eukaryotic cell lines
☒	☐	Palaeontology and archaeology
☐	☒	Animals and other organisms
☐	☒	Human research participants
☒	☐	Clinical data
☒	☐	Dual use research of concern

Methods

n/a	Involved in the study	
☒	☐	ChIP-seq
☒	☐	Flow cytometry
☒	☐	MRI-based neuroimaging

Antibodies

Antibodies used	
Antibodies used	-Merlin (#ab88957, clone AF1G4, Abcam, 1:2000)<br>-GAPDH (#MA515738, clone GA1R, Thermo Fischer Scientific, 1:2000)<br>-Caspase-7 (#9492, Cell Signaling, 1:500)<br>-IRF8 (#5628S, clone D20D8, Cell Signaling, 1:500)<br>-Tubulin (#T5168, clone B-5-1-2, Sigma, 1:5000)<br>-HH3 (#702023, clone 17H2L9, Thermo Fischer Scientific, 1:1000)<br>-FLAG (#F1804, clone F1804, Sigma, 1:1000)<br>-ARHGAP35 (#2860, clone C59F7, Cell Signaling, 1:1000)<br>-FOXM1 (#sc-376471, clone G-5, Santa Cruz, 1:500)<br>-pRB-S780 (#8180P, clone D59B7, Cell Signaling, 1:1000)<br>-pRB-S807/811 (#8516P, clone D20B12, Cell Signaling, 1:1000)<br>-Anti-mouse HRP-conjugated secondary antibody (#7076, Cell Signaling, 1:2000)<br>-Anti-rabbit HRP-conjugated secondary antibody (#7074, Cell Signaling, 1:2000)<br>-LYVE-1 (#ab14917, Abcam, 1:1000)<br>-PROX-1 (#AF2727, R&D Systems, 1:1000)<br>-Anti-rabbit Alexa Fluor secondary antibody (#A21206, Thermo Fischer Scientific, 1:1000)<br>-Anti-goat Alexa Fluor secondary antibody (#A21469, Thermo Fischer Scientific 1:1000)<br>-FOXM1 (#ab207298, clone EPR17379, Abcam, 1:600)<br>-Ki-67 (#790-4286, clone 30-9, Ventana, 1:6)<br>-cleaved Caspase-3 (#9664, clone 5A1E, Cell Signaling, 1:2000)<br>-CD3 (#A0452, Agilent Technologies, 1:200)
Validation	-Merlin: Knockout validated for human immunoblots.<br>-GAPDH: Knockout validated for human immunoblots.<br>-Caspase-7: Validated +/- apoptosis induction for human immunoblots.<br>-IRF8: Validated +/- IFN stimulation for human immunoblots. An unknown background band is detected at 80 kDa in some cell lines.<br>-Tubulin: Validated for human immunoblots using recombinant expressed antibodies, genetic strategies, independent antibody verification, RNA sequencing, functional assays, expression/overexpression, and immunocapture followed by mass spectrometry.<br>-HH3: Validated for human immunoblots using subcellular fractionation.<br>-FLAG: Validated for human immunoblots and immunoprecipitation using affinity purification and competition assays.<br>-ARHGAP35: Validated for human immunoblots using immunoprecipitation<br>-FOXM1: Validated for human immunoblots using overexpression<br>-pRB-S780: Validated for human immunoblots using phosphorylated or nonphosphorylated recombinant truncated Rb with or without RB blocking peptides.<br>-pRB-S807/811 : Validated for human immunoblots using phosphorylated or nonphosphorylated recombinant truncated Rb with or without RB blocking peptides.<br>-Anti-mouse HRP-conjugated secondary antibody: Validated for human immunoblots using affinity purification and competition assays.<br>-Anti-rabbit HRP-conjugated secondary antibody: Validated for human immunoblots using affinity purification and competition assays.<br>-LYVE-1: Knockout validated for human imunofluorescence.<br>-PROX-1: Validated for human immunofluorescence using subcellular localization.<br>-Anti-rabbit Alexa Fluor secondary antibody: Validated for human immunofluorescence using affinity purification.<br>-Anti-goat Alexa Fluor secondary antibody: Validated for human immunofluorescence using affinity purification.<br>-FOXM1: Validated for human immunofluorescence using recombinant expressed antibodies.<br>-Ki-67: Validated for human immunohistochemistry and immunofluorescence using proliferating versus non-proliferating tissues.<br>-cleaved Caspase-3: Validated +/- apoptosis induction for human immunohistochemistry<br>-CD3: Validated for human immunohistochemistry using T-cell versus B-cell lines.

Eukaryotic cell lines

Policy information about cell lines

Cell line source(s)	HEK293T cells were obtained from ATCC. CH-157MN, IOMM-Lee, DI-98, DI-134, MSC1, and M10G primary meningioma cell lines were obtained from collaborators or derived from patient tumor samples and described in previous studies, as referenced in the Methods section.
Authentication	Meningioma cell lines were authenticated using DNA methylation profiling and CNV analyses to confirm concordance to tumors of origin, most recently in 2021. Non-meningioma cell lines purchased from reputable commercial suppliers (HEK293T cells from ATCC) were not authenticated.
Mycoplasma contamination	All cell lines tested negative for mycoplasma.
Commonly misidentified lines (See ICLAC register)	No commonly misidentified cell lines were used in this study.

Animals and other organisms

Policy information about studies involving animals; ARRIVE guidelines recommended for reporting animal research

Laboratory animals	5-6 week old female NU/NU mice purchased from Harlan Sprague Dawley for this study. All animal care and experimental procedures were in accordance with federal policies and guidelines governing the use of animals and were approved by the University of California San Francisco's (UCSF) Institutional Animal Care and Use Committee (IACUC). The IACUC is in full compliance with the 8th edition of The Guide for the Care and use of Laboratory Animals. UCSF has an AAALAC accredited animal care and use program. Mice were housed in solid-bottomed cages containing autoclaved paper chips in individually ventilated cages. Animals had continuous access to irradiated food and water purified by reverse osmosis and UV lighting. The housing room was maintained at 68 to 74º Fahrenheit with 30-70 % relative humidity. All cages were maintained in a SPF barrier facility from which dirty bedding sentinel mice were tested quarterly. All sentinels were found to be seronegative for mouse hepatitis virus, pneumonia virus of mice, mouse parvovirus, minute virus of mice, epizootic diarrhea of infant mice, Theiler's murine encephalomyelitis virus, ectromelia and were free of ectoparasites and endoparasites. Mice were observed daily by animal care staff for any clinical abnormalities.
Wild animals	Study did not involve wild animals.
Field-collected samples	Study did not involve samples collected in the field.
Ethics oversight	Study was approved by the UCSF Institutional Animal Care and Use Committee (AN174769).

Note that full information on the approval of the study protocol must also be provided in the manuscript.

Human research participants

Policy information about studies involving human research participants

Population characteristics	Patients undergoing resection of meningioma at UCSF or HKU of all ages, genders, past and current diagnosis and treatment categories were included. Covariates are summarized in Supplementary table 1, and are recapitulated here:<br><br>Patients: 565<br>Median age: 58 years<br>Median follow-up: 5.6 years<br>Male:Female (ratio): 193:372 (1:1.93)<br>Recurrences: 161<br>Extent of resection<br>Gross total: 394 (70%)<br>Near total: 171 (30%)<br>WHO grade<br>1 : 388 (69%)<br>2 (atypical): 142 (25%)<br>3 (anaplastic): 35 (6%)
Recruitment	As part of routine clinical practice at UCSF and HKU institutions, all patients undergoing craniotomy for tumor resection sign a waiver of informed consent to contribute de-identified data to research projects. Thus, there was no self-selection bias or other biases that may influence or impact our results. Meningioma samples for the discovery cohort were selected from the UCSF Brain Tumor Center Biorepository and Pathology Core in 2017, with an emphasis on high-grade meningiomas and low-grade meningiomas with long clinical follow-up. All WHO grade 2 and grade 3 meningiomas with available frozen samples were included. For WHO grade 1 meningiomas, frozen samples in the tissue bank were cross-referenced for clinical follow-up data from a retrospective institutional meningioma clinical outcomes database, and all cases with available frozen tissue and clinical follow-up greater than 10 years (n=40) were included. To achieve a discovery cohort of 200 cases, additional WHO grade 1 meningiomas with available frozen tissue and the longest possible clinical follow-up (albeit less than 10 years, n=47) were included. The electronic medical record was reviewed for all patients in late 2018, and paper charts were reviewed in early 2019 for patients treated prior to the advent of the electronic medical record. All available clinical pathology material was reviewed for diagnostic accuracy by a board-certified neuropathologist (D.A.S.). WHO grading was performed using contemporary criteria outlined in the WHO classification of tumors of the central nervous system. Cases for which other tumors remained in the differential diagnosis (such as schwannoma or solitary fibrous tumor/hemangiopericytoma) were excluded. The validation cohort was comprised of 365 consecutive meningiomas from patients who were treated at The University of Hong Kong (HKU) from 2000 to 2019 that had frozen tissue suitable for DNA methylation profiling. The medical record was reviewed for all patients in late 2019. For the discovery and validation cohorts, meningioma recurrence was defined as new radiographic tumor on magnetic resonance imaging after gross total resection, or enlargement/progression/growth of residual tumor on magnetic resonance imaging after subtotal resection. All magnetic resonance imaging studies in the discovery cohort were reviewed for accuracy and meningioma location by a board-certified radiologist with a Certificate of Added Qualification in Neuroradiology (J.E.V-M.) (Supplementary note). Nomograms integrating clinical and molecular features influencing meningioma outcomes were developed to guide clinical translation of meningioma DNA methylation groups (Supplementary note).
Ethics oversight	This study complied with all relevant ethical regulations and was approved by the UCSF Institutional Review Board (IRB #13-12587, #17-22324, #17-23196, and #18-24633), and by the HKU Institutional Review Board (UW 07-273 and UW 21-112). Meningiomas and de-identified clinical information were transferred from HKU to UCSF in 2019 for analysis under protection of a Material Transfer Agreement that was certified by both institutions.

Note that full information on the approval of the study protocol must also be provided in the manuscript.
